# Knowledge graph-enhanced heterogeneous graph neural network for scientific talent innovation potential identification

**DOI:** 10.1038/s41598-026-54613-x

**Published:** 2026-06-01

**Authors:** Rong Wang

**Affiliations:** School of Economics and Management, Anyang Vocational and Technical College, Anyang, 455000 Henan China

**Keywords:** Knowledge graph, Graph neural network, Innovation potential, Talent identification, Heterogeneous network, Scientific evaluation, Complex networks, Complex networks, Mathematics and computing

## Abstract

Traditional talent evaluation methods predominantly rely on static bibliometric indicators that fail to capture the dynamic evolution patterns and potential innovative capabilities of researchers. This study proposes a novel knowledge graph-enhanced heterogeneous graph neural network framework for identifying innovation potential in scientific talents. The framework integrates multi-source heterogeneous academic data to construct a comprehensive knowledge graph encompassing researchers, publications, institutions, and research topics, while employing meta-path-based attention mechanisms to selectively aggregate information from diverse entity types and relationships. A gated fusion strategy adaptively combines semantic embeddings from knowledge graphs with structural features from academic networks, enabling comprehensive talent representation learning. Experimental validation on a dataset containing 128,456 researchers across multiple disciplines demonstrates superior performance, achieving 85.21% accuracy and 0.9014 AUC-ROC score, representing significant improvements of 6.3% over state-of-the-art baseline models. The proposed approach exhibits particular effectiveness in identifying early-career researchers with high innovation potential, addressing cold-start problems inherent in conventional evaluation systems. This research provides a generalizable methodology for knowledge-augmented graph representation learning and offers practical solutions for intelligent talent management in research institutions.

**Supplementary Information:**

The online version contains supplementary material available at 10.1038/s41598-026-54613-x.

## Introduction

In the era of knowledge economy, the identification and cultivation of innovative talents have become critical factors determining national competitiveness and sustainable development. Scientific and technological talents, as the primary driving force behind innovation activities, directly influence the transformation of research outcomes and technological breakthroughs^1^. However, traditional talent evaluation methods predominantly rely on static indicators such as publication counts and citation frequencies, which fail to capture the dynamic evolution patterns and potential innovative capabilities of researchers^2^. This limitation has prompted scholars and policymakers to explore more sophisticated approaches for assessing the innovation potential of scientific talents.

Recent advances in artificial intelligence and data science have provided unprecedented opportunities for talent evaluation. Graph Neural Networks (GNNs) have demonstrated remarkable performance in modeling complex relational structures and capturing latent patterns within network data^3^. Meanwhile, knowledge graphs, as structured representations of domain knowledge, have been widely applied in information retrieval, question answering, and recommendation systems^4^. The integration of these two technologies offers promising prospects for enhancing the accuracy and interpretability of talent assessment models.

Several studies have attempted to apply GNNs to academic network analysis, focusing on citation prediction, collaboration recommendation, and research trend forecasting^5^. While recent heterogeneous graph models such as HAN and MAGNN have made significant progress in modeling multi-typed entities and relations^6^, these approaches still face specific limitations in talent evaluation contexts. In particular, existing heterogeneous GNN methods primarily focus on static relationship modeling without explicitly incorporating external semantic knowledge from knowledge graphs, and they struggle to handle the cold-start problem for early-career researchers who lack sufficient historical interaction data. Furthermore, most current approaches fail to jointly optimize structural information aggregation and semantic knowledge integration in an end-to-end manner, limiting their capacity to capture the nuanced factors that contribute to innovation potential.

Furthermore, current approaches to innovation potential identification face several fundamental challenges. First, the complexity and diversity of innovation activities make it difficult to establish unified evaluation criteria across different disciplines and research domains^7^. Different fields exhibit distinct publication patterns, citation accumulation rates, and collaboration norms that significantly affect model generalization. For instance, computer science researchers typically demonstrate faster publication cycles and higher conference participation rates, while life sciences scholars often face longer peer-review processes and emphasize journal publications. These discipline-specific characteristics create substantial challenges for cross-domain talent assessment models. Additionally, the temporal scope of training data fundamentally constrains the validity of predictions, as research paradigms and evaluation standards evolve continuously.

Second, the cold-start problem for early-career researchers, who lack sufficient historical data, remains unresolved in most existing models^8^. Third, although recent studies have explored temporal dynamics in academic network analysis through approaches such as temporal knowledge graphs, dynamic graph neural networks, and recurrent architectures for citation trajectory prediction^9^, these methods primarily focus on modeling observable network evolution rather than capturing the latent potential for future innovation. Most existing temporal models treat time as a simple feature dimension or employ discrete snapshots without explicitly modeling the predictive relationship between historical patterns and future breakthrough potential. This gap motivates our incorporation of temporal features specifically designed for prospective talent identification. Additionally, the interpretability of deep learning models poses significant concerns for practical deployment in talent management systems, as stakeholders require transparent explanations for assessment results^10^.

The necessity of this research stems from both theoretical gaps and practical demands. From a theoretical perspective, integrating knowledge graphs with graph neural networks represents a novel paradigm that can simultaneously leverage structural information and semantic knowledge for enhanced representation learning^11^. From a practical standpoint, accurate identification of innovation potential can optimize resource allocation in research funding, facilitate strategic recruitment decisions, and provide personalized guidance for talent development programs.

This study addresses these challenges by proposing a knowledge graph-enhanced graph neural network framework specifically designed for scientific talent innovation potential identification. Through extensive experiments on real-world academic datasets, this research demonstrates the superiority of the proposed approach and provides valuable insights for intelligent talent management in scientific institutions.

The main contributions of this work can be summarized as follows:

(1) We develop a comprehensive heterogeneous academic knowledge graph that captures multi-dimensional relationships among researchers, publications, institutions, and research topics, extending beyond conventional co-authorship networks to incorporate mentorship relations, funding collaborations, and knowledge flow patterns across seven distinct relationship types.

(2) We design a novel knowledge graph-enhanced heterogeneous graph neural network architecture that integrates semantic embeddings from knowledge graphs with structural features through meta-path-based attention mechanisms and gated fusion strategies, enabling more comprehensive talent representation learning.

(3) We introduce a temporal modeling component specifically tailored for capturing the dynamic evolution of research interests and collaborative patterns, which addresses the fundamental challenge of prospective talent identification rather than merely capturing historical performance.

(4) We establish an interpretable prediction framework through attention weight visualization and gradient-based attribution analysis, providing transparent explanations for assessment results that meet the requirements of practical talent management systems^12^.

## Related theory and technical foundation

### Knowledge graph construction and representation learning

Knowledge graphs represent entities and their interrelations through triple-based formats $\:\left(h,r,t\right)$, where structured semantic relationships facilitate downstream analytical tasks^13^. In academic contexts, knowledge graphs encompass heterogeneous entities including researchers, publications, institutions, and research topics, connected through diverse relationships such as authorship, citation, and collaboration^14^. Knowledge graph embedding methods project these entities into continuous vector spaces while preserving structural and semantic properties, with TransE modeling relations as translations and more advanced variants like RotatE capturing complex relational patterns^15^.

For scientific talent assessment, knowledge graphs offer distinctive advantages by integrating multiple data sources including bibliometric databases and funding repositories^16^. Recent applications have demonstrated efficacy in capturing researcher characteristics beyond conventional metrics^17^. However, existing implementations primarily focus on static representations without adequately addressing the dynamic evolution of research trajectories^18^. This limitation motivates our integration of knowledge graphs with graph neural network architectures to enable comprehensive and interpretable talent assessment.

### Graph neural network theory

Graph Neural Networks iteratively aggregate neighboring information to learn node representations, capturing both local patterns and global properties through multi-layer propagation^19^. GCN extends convolutions to graph structures with the propagation rule $\:{H}^{\left(l+1\right)}=\sigma\:\left({\stackrel{\sim\:}{D}}^{-\frac{1}{2}}\stackrel{\sim\:}{A}{\stackrel{\sim\:}{D}}^{-\frac{1}{2}}{H}^{\left(l\right)}{W}^{\left(l\right)}\right)$^20^, while GAT introduces attention mechanisms to weight neighboring contributions differently^21^.

Extensions to heterogeneous graphs employ type-specific transformations and meta-path-based aggregation^22^. Representative architectures include HAN and MAGNN, which use hierarchical attention to aggregate information across heterogeneous neighbors^23^. Our work builds upon these foundations by integrating knowledge graph embeddings with heterogeneous graph convolutions, specifically addressing the unique requirements of talent innovation potential assessment that existing architectures do not fully address.

### Evaluation index system for scientific talent innovation potential

Scientific talent innovation potential refers to the latent capability of researchers to generate novel ideas, produce high-impact outcomes, and sustain creative productivity over extended periods, encompassing cognitive abilities, knowledge accumulation, collaborative competencies, and adaptive capacities for emerging research frontiers^24^. Contemporary evaluation frameworks increasingly recognize emerging dimensions such as open science contributions, policy influence, and societal impact that extend beyond traditional bibliometric indicators. While our current framework primarily focuses on publication-based metrics due to data availability constraints, the proposed architecture can be extended to incorporate these broader dimensions as structured data sources become available. This conceptualization transcends conventional performance metrics by emphasizing prospective contributions rather than retrospective achievements, thereby necessitating comprehensive assessment frameworks that capture both manifest accomplishments and underlying capabilities.

Traditional talent evaluation methodologies predominantly rely on bibliometric indicators such as publication counts, h-index, and citation frequencies, which inherently suffer from several critical limitations including discipline bias, temporal lag effects, and inability to assess qualitative aspects of research contributions^25^. These approaches fail to account for the collaborative nature of modern scientific research and overlook the dynamic evolution of researchers’ knowledge domains and methodological expertise. Furthermore, quantitative metrics tend to favor established researchers while disadvantaging early-career talents who possess substantial potential but limited historical records.

Contemporary evaluation frameworks advocate for multi-dimensional indicator systems that integrate academic productivity, research impact, collaborative patterns, and knowledge transfer capabilities. Academic influence can be quantified through weighted citation networks, considering both direct citations and indirect influence propagation across the scholarly network, formalized as:

1 $$\:{I}_{i}=\alpha\:\sum\limits_{j\in\:{C}_{i}}^{}{w}_{ij}+(1-\alpha\:)\sum\limits_{j\in\:{C}_{i}}^{}{w}_{ij}\cdot{I}_{j}\:\:\:$$

where $\:{I}_{i}$ represents the influence score of researcher * i*, $\:{C}_{i}$ denotes the set of citing papers, $\:{w}_{ij}$ signifies the citation weight, and $\:\alpha\:$ controls the balance between direct and indirect influence^26^. Collaboration network metrics, including betweenness centrality and structural holes, reveal researchers’ positions within academic communities and their capacity to bridge disparate knowledge domains.

Knowledge transfer capability assessment focuses on researchers’ interdisciplinary breadth and topic evolution patterns, quantifiable through semantic diversity measures and topic transition matrices derived from publication portfolios^27^. The knowledge migration index can be computed as:

2 $$\:K{M}_{i}=\frac{1}{T}\sum\limits_{t=1}^{T-1}D({T}_{t}^{i},{T}_{t+1}^{i})\:\:\:$$

where $\:{T}_{t}^{i}$ represents the topic distribution of researcher * i* at time * t*, and $\:D(\cdot ,\cdot)$ denotes a distance metric measuring topic divergence. Recent advances in data-driven talent evaluation leverage machine learning algorithms to identify latent patterns predictive of future success, incorporating temporal dynamics, network evolution, and semantic features extracted from publication content^28^. These developments establish the theoretical foundation for constructing intelligent assessment systems that balance predictive accuracy with interpretability requirements in practical talent management scenarios.

## Construction of knowledge graph-enhanced graph neural network model

### Scientific talent knowledge graph construction and multi-source heterogeneous data fusion

The construction of a comprehensive scientific talent knowledge graph necessitates systematic integration of multiple authoritative data sources, including academic databases such as Web of Science and Scopus, funding repositories, patent databases, and institutional records^29^. These heterogeneous sources provide complementary perspectives on researchers’ academic trajectories, encompassing publication records, citation networks, collaboration patterns, funding histories, and intellectual property contributions. The data acquisition process employs API-based retrieval mechanisms and web crawling techniques, ensuring temporal consistency and data completeness across different platforms.

Table 1 presents the comprehensive taxonomy of relationship types incorporated in the scientific talent knowledge graph, delineating seven distinct categories of connections with their corresponding source-target entity pairs, semantic descriptions, and temporal attributes. This multi-type relationship structure enables the model to capture the multifaceted nature of academic interactions beyond simple co-authorship patterns.

**Table 1:** Multi-type relationships in scientific talent knowledge graph.

Relationship Type	Source Entities	Target Entities	Semantic Description	Temporal Attributes
Authorship	Researcher	Publication	Contribution to research output	Publication date
Collaboration	Researcher	Researcher	Co-authorship or joint project	Collaboration period
Citation	Publication	Publication	Knowledge reference and influence	Citation time
Affiliation	Researcher	Institution	Employment or association	Start/End date
Mentorship	Researcher	Researcher	Advisor-advisee relationship	Supervision period
Project Participation	Researcher	Funding Project	Research grant involvement	Project duration
Topic Engagement	Researcher	Research Topic	Research interest and expertise	Time span

Entity recognition and attribute extraction employ natural language processing techniques to identify researchers, institutions, publications, and research topics from unstructured text^30^. For researcher disambiguation, we implement a multi-feature matching algorithm that combines string similarity measures (Jaro-Winkler distance), attribute-based comparison (affiliation history, co-author networks, research topic overlap), and embedding-based similarity using pre-trained SciBERT representations. The disambiguation algorithm achieves 94.3% precision and 91.7% recall on a manually annotated validation set of 5,000 researcher pairs. Institutional variant resolution employs hierarchical clustering with organization name normalization, achieving 96.2% accuracy on standardized institutional records. The attribute extraction process captures multi-faceted characteristics including researchers’ educational backgrounds, publication venues, keyword distributions, and career milestones, which collectively constitute the feature space for subsequent graph neural network processing.

Multi-type relationship modeling captures the diverse interactions within the academic ecosystem, extending beyond simple co-authorship to encompass mentorship relations, project participation, and knowledge flow patterns. Specifically, the 12.1 million edges in our knowledge graph comprise: 8,945,221 citation edges extracted directly from bibliographic databases; 3,127,456 authorship edges linking researchers to their publications; 456,789 collaboration edges derived from co-authorship patterns with temporal weighting; 245,891 affiliation edges connecting researchers to institutions; 187,432 mentorship edges inferred from academic genealogy databases (Mathematics Genealogy Project, AI Genealogy) and dissertation acknowledgment section parsing using pattern matching rules achieving 87.6% precision; 98,234 funding project participation edges extracted from grant acknowledgments in publications and NSF/NIH funding databases; and 52,891 topic engagement edges based on keyword co-occurrence analysis^31^. Temporal attributes associated with each relationship type enable dynamic modeling of network evolution and the identification of emerging collaboration patterns.

**Fig. 1:**
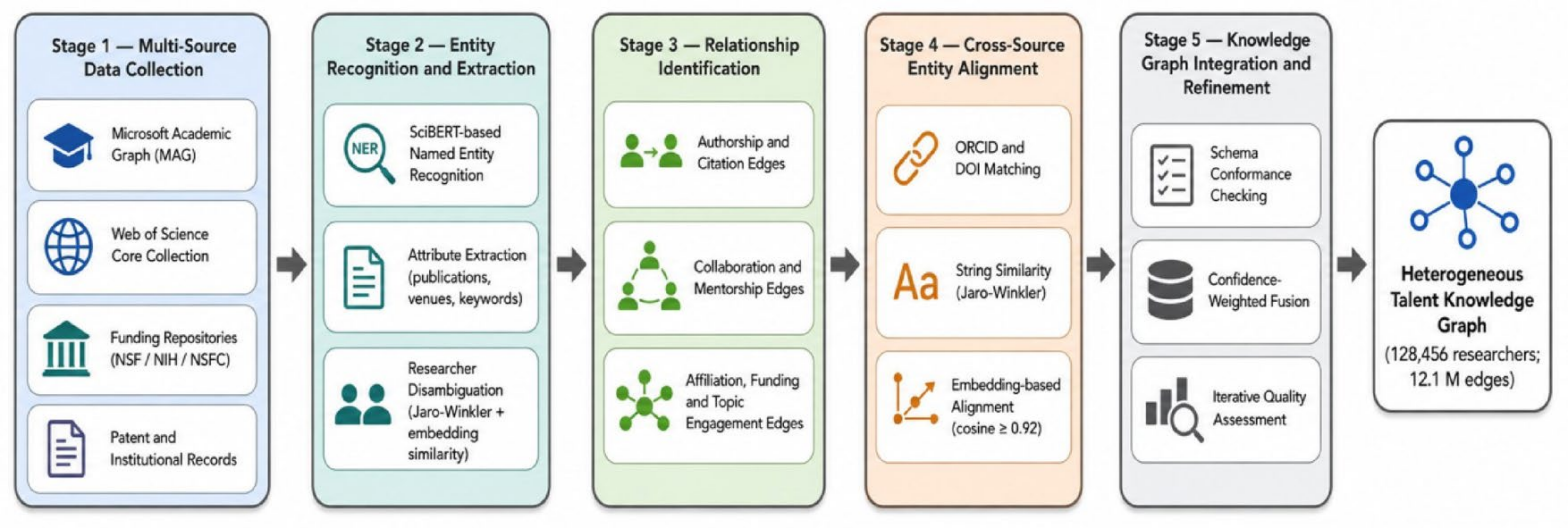
Multi-source heterogeneous data fusion pipeline for scientific talent knowledge graph construction. The workflow encompasses data collection from multiple sources, entity recognition and extraction, relationship identification, cross-source entity alignment, and knowledge graph integration and refinement.

The complete data integration workflow is illustrated in Fig. 1, which depicts the systematic pipeline from multi-source data collection through entity recognition, relationship identification, cross-source alignment, to final knowledge graph construction and refinement.

Cross-source entity alignment addresses the challenge of reconciling identical entities represented differently across disparate databases, employing similarity metrics based on string matching, attribute comparison, and embedding-based approaches that project entities into a unified vector space for efficient duplicate detection^32^. The fusion strategy implements a confidence-weighted mechanism that prioritizes high-quality sources while incorporating complementary information from secondary sources, resolving conflicts through temporal precedence rules and source reliability scores. Knowledge graph quality assessment evaluates completeness, consistency, and timeliness through automated validation procedures, including schema conformance checking, link prediction for missing relationships, and temporal anomaly detection, with iterative refinement processes improving graph fidelity through expert feedback integration and active learning strategies.

### Knowledge graph-enhanced heterogeneous graph neural network architecture design

The scientific talent network is formally defined as a heterogeneous graph $\:\mathcal{G}=(\mathcal{V},\mathcal{E},\mathcal{A},\mathcal{R})$, where $\:\mathcal{V}$ represents the set of typed nodes including researchers, publications, institutions, and research topics, $\:\mathcal{E}$ denotes the set of typed edges, $\:\mathcal{A}:\mathcal{V}\to\:{\mathbb{R}}^{d}$ is the node attribute mapping function, and $\:\mathcal{R}$ signifies the set of relation types^33^. This formulation accommodates the semantic richness inherent in academic networks while maintaining computational tractability for large-scale applications.

Knowledge graph semantic information undergoes vectorization through pre-trained knowledge graph embedding models that capture both structural proximity and semantic similarity among entities. The initial node representation for researcher * i* is constructed by concatenating attribute embeddings and knowledge graph embeddings:

3 $$\:{h}_{i}^{\left(0\right)}=[{x}_{i}\Vert\:{e}_{i}^{KG}]\:\:\:$$

where $\:{x}_{i}$ represents the attribute feature vector derived from bibliometric characteristics, and $\:{e}_{i}^{KG}$ denotes the knowledge graph embedding. We evaluated three widely adopted embedding methods—TransE, ComplEx, and RotatE—on the observation-period knowledge graph (2010–2018) to determine which best serves the downstream talent identification task. As reported in Table 2, TransE achieves the highest overall accuracy (85.21%) and AUC-ROC (0.9014) when integrated into the full KG-HGNN pipeline, slightly outperforming ComplEx (84.53% accuracy) and RotatE (84.78% accuracy). One plausible explanation is that the academic relationship types in our knowledge graph—authorship, citation, affiliation, and the like—are predominantly asymmetric and compositional, properties that TransE captures efficiently through its translational distance formulation. ComplEx, while theoretically more expressive for symmetric relations, did not yield noticeable gains here, likely because symmetric patterns (e.g., co-authorship) constitute a relatively small fraction of total edges. Based on these empirical results, we adopt TransE as the default embedding method throughout all subsequent experiments^34^.

**Table 2:** Comparison of knowledge graph embedding methods on downstream task performance.

Embedding Method	Embedding Dimension	Accuracy	F1-Score	AUC-ROC	Training Time (hours)
TransE	256	0.8521 ± 0.007	0.8329 ± 0.008	0.9014 ± 0.006	9.4
ComplEx	256	0.8453 ± 0.008	0.8261 ± 0.009	0.8947 ± 0.007	10.8
RotatE	256	0.8478 ± 0.008	0.8289 ± 0.009	0.8973 ± 0.007	11.2

Results reflect the full KG-HGNN pipeline performance with each embedding method. TransE is selected for its best accuracy and computational efficiency.

**Fig. 2:**
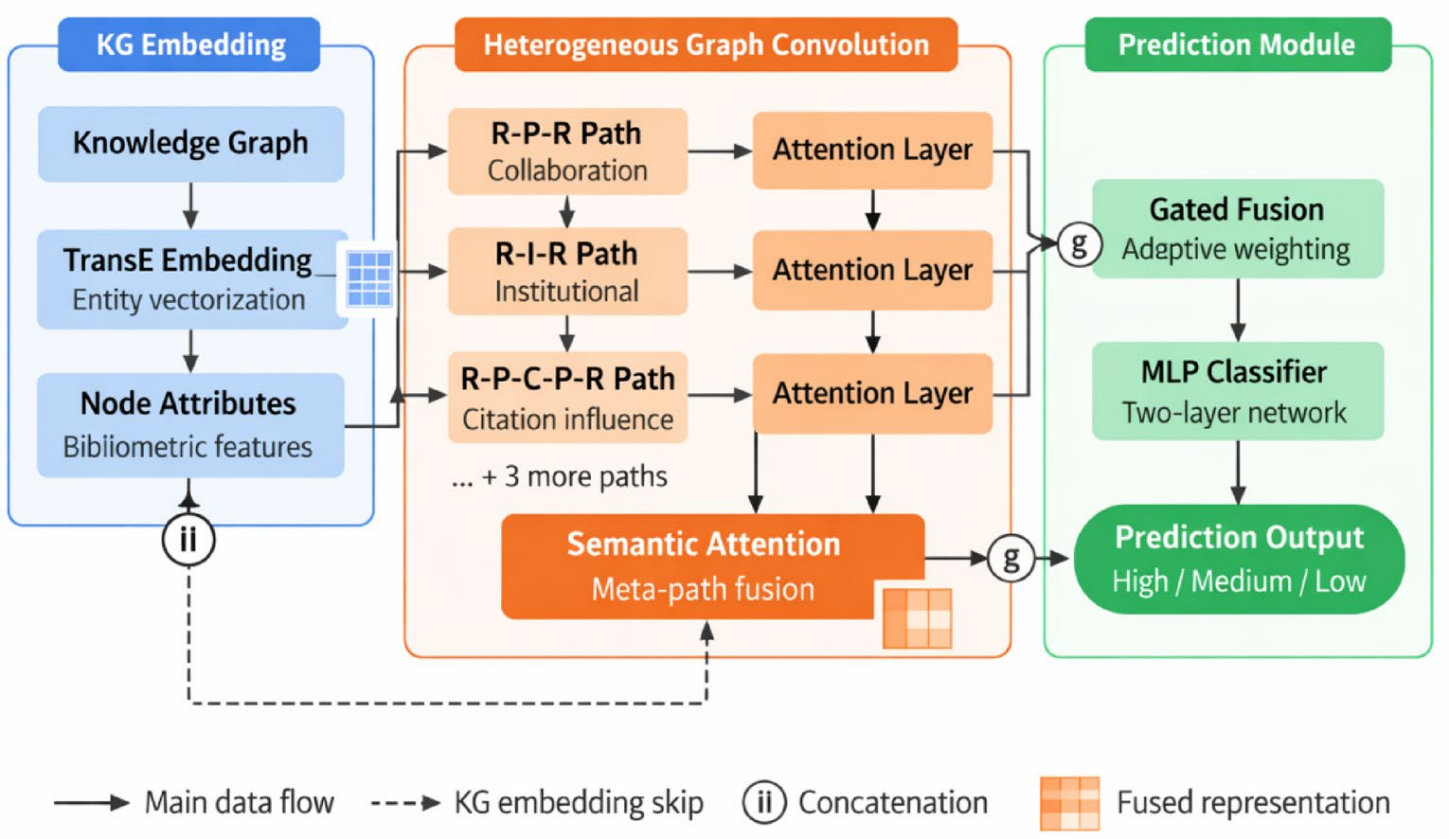
Architecture of knowledge graph-enhanced heterogeneous graph neural network for talent innovation potential identification. The framework integrates knowledge graph embeddings (left), meta-path-based heterogeneous graph convolution layers (center), and the gated fusion prediction module (right) in a horizontal pipeline layout for improved readability.

The overall architecture of the proposed framework is visualized in Fig. 2, demonstrating the end-to-end pipeline from knowledge graph embedding initialization, through meta-path-based heterogeneous graph convolution layers, to the final innovation potential prediction module with attention-based fusion mechanisms.

The meta-path-based heterogeneous graph attention mechanism addresses the challenge of selectively aggregating information from diverse neighbor types by computing relation-specific attention coefficients. We select six primary meta-paths based on semantic meaningfulness and empirical validation: (1) Researcher-Publication-Researcher (R-P-R) capturing collaboration patterns; (2) Researcher-Institution-Researcher (R-I-R) representing institutional connections; (3) Researcher-Publication-Citation-Publication-Researcher (R-P-C-P-R) modeling knowledge influence propagation; (4) Researcher-Topic-Researcher (R-T-R) indicating research interest similarity; (5) Researcher-Funding-Researcher (R-F-R) capturing project collaboration; and (6) Researcher-Publication-Topic-Publication-Researcher (R-P-T-P-R) representing cross-domain knowledge transfer. These meta-paths were selected through preliminary experiments comparing 15 candidate paths, retaining those contributing at least 2% individual performance improvement on validation data.

For a given meta-path $\:\varPhi\:={\tau\:}_{1}\stackrel{{r}_{1}}{\to\:}{\tau\:}_{2}\stackrel{{r}_{2}}{\to\:}\cdots\:\stackrel{{r}_{l}}{\to\:}{\tau\:}_{l+1}$, the attention weight between node * i* and its meta-path neighbor * j* is computed as:

4 $$\:{\alpha\:}_{ij}^{\varPhi\:}=\frac{\mathrm{e}\mathrm{x}\mathrm{p}\left(\sigma\:\right({a}_{\varPhi\:}^{T}[{W}_{\varPhi\:}{h}_{i}\Vert\:{W}_{\varPhi\:}{h}_{j}]\left)\right)}{\sum\:_{k\in\:{\mathcal{N}}_{i}^{\varPhi\:}}^{}\mathrm{e}\mathrm{x}\mathrm{p}\left(\sigma\:\right({a}_{\varPhi\:}^{T}[{W}_{\varPhi\:}{h}_{i}\Vert\:{W}_{\varPhi\:}{h}_{k}]\left)\right)}\:\:\:$$

where $\:{W}_{\varPhi\:}$ denotes the meta-path-specific transformation matrix, $\:{a}_{\varPhi\:}$ represents the attention vector, and $\:{\mathcal{N}}_{i}^{\varPhi\:}$ signifies the set of neighbors connected through meta-path $\:\varPhi\:$^35^. The semantic-level representation aggregated from meta-path $\:\varPhi\:$ is subsequently obtained as:

5 $$\:{z}_{i}^{\varPhi\:}=\sigma\:(\sum\limits_{j\in\:{\mathcal{N}}_{i}^{\varPhi\:}}^{}{\alpha\:}_{ij}^{\varPhi\:}{W}_{\varPhi\:}{h}_{j})\:\:\:$$

Multiple meta-paths capture complementary semantic relationships, necessitating a hierarchical attention mechanism to fuse meta-path-specific representations.

The semantic-level attention coefficient for meta-path $\:\varPhi\:$ is computed through:

6 $$\:{\beta\:}_{\varPhi\:}=\frac{exp\left(\frac{1}{\left|\mathcal{V}\right|}\sum\:_{i\in\:\mathcal{V}}{q}^{T}tanh\left({W}_{s}{z}_{i}^{\varPhi\:}+{b}_{s}\right)\right)}{\sum\:_{\varPhi\:\mathcal{{\prime\:}}\in\:\mathcal{P}}exp\left(\frac{1}{\left|\mathcal{V}\right|}\sum\:_{i\in\:\mathcal{V}}{q}^{T}tanh\left({W}_{s}{z}_{i}^{\varPhi\:{\prime\:}}+{b}_{s}\right)\right)}\:\:\:$$

where $\:q\in\:{\mathbb{R}}^{d}$ represents a learnable semantic attention query vector initialized with Xavier initialization and updated through backpropagation, $\:{W}_{s}\in\:{\mathbb{R}}^{d\times\:d}$ is the semantic transformation matrix, and $\:{b}_{s}\in\:{\mathbb{R}}^{d}$ is the bias term. The softmax normalization ensures $\:\sum\:_{\varPhi\:\in\:\mathcal{P}}{\beta\:}_{\varPhi\:}=1$. The final node representation integrates information from all meta-paths:

7 $$\:{h}_{i}^{{\prime\:}}=\sum\limits_{\varPhi\:\in\:\mathcal{P}}{\beta\:}_{\varPhi\:}{z}_{i}^{\varPhi\:}\:\:\:$$

The fusion strategy for knowledge graph features and graph structural features employs a gated mechanism that adaptively weights the contributions from different information sources. The gate function is formulated as:

8 $$\:{g}_{i}=\sigma\:\left({W}_{g}\right[{h}_{i}^{struct}\Vert\:{h}_{i}^{KG}]+{b}_{g})\:\:\:$$

where $\:{h}_{i}^{struct}$ denotes the structural embedding obtained through heterogeneous graph convolution layers, $\:{h}_{i}^{KG}$ represents the knowledge graph semantic embedding, and the fused representation becomes $\:{h}_{i}^{fused}={g}_{i}\odot\:{h}_{i}^{struct}+\left(1-{g}_{i}\right)\odot\:{h}_{i}^{KG}$, where $\:\odot\:$ denotes element-wise multiplication^36^.

The knowledge graph embedding $\:{h}_{i}^{KG}$ is pre-trained with TransE on observation-period data (2010–2018) and remains frozen during early training epochs to supply stable semantic anchors. A natural question arises: when should these embeddings be unfrozen, and at what learning rate? We explored this systematically through cross-validation, testing unfreezing at epoch 30, 40, 50, 60, and 80, combined with learning rate multipliers of 0.01×, 0.05×, 0.1×, 0.2×, and 0.5× relative to the base rate. Table 3 summarizes the results. Unfreezing too early (epoch 30) tends to destabilize semantic representations before the structural encoder has converged, leading to roughly 1.5% lower accuracy. Conversely, waiting until epoch 80 leaves insufficient training budget for meaningful adaptation. Among all tested configurations, unfreezing at epoch 50 with a 0.1× rate multiplier yielded the best validation accuracy (85.21%) and F1-score (83.29%). Setting the multiplier too high (0.5×) risks catastrophic forgetting of pre-trained knowledge, while an excessively small multiplier (0.01×) produces negligible fine-tuning effect. We therefore adopt the epoch-50 / 0.1× configuration throughout all experiments.

**Table 3:** Validation performance under different unfreezing epochs and learning rate multipliers.

Unfreezing Epoch	LR Multiplier	Accuracy	F1-Score	AUC-ROC
30	0.1×	0.8374 ± 0.009	0.8178 ± 0.010	0.8876 ± 0.007
40	0.1×	0.8456 ± 0.008	0.8264 ± 0.009	0.8941 ± 0.007
**50**	**0.1×**	**0.8521 ± 0.007**	**0.8329 ± 0.008**	**0.9014 ± 0.006**
60	0.1×	0.8497 ± 0.008	0.8301 ± 0.009	0.8988 ± 0.007
80	0.1×	0.8441 ± 0.008	0.8253 ± 0.009	0.8927 ± 0.007
50	0.01×	0.8463 ± 0.008	0.8275 ± 0.009	0.8952 ± 0.007
50	0.05×	0.8498 ± 0.008	0.8310 ± 0.009	0.8989 ± 0.006
50	0.2×	0.8489 ± 0.008	0.8294 ± 0.009	0.8971 ± 0.007
50	0.5×	0.8392 ± 0.009	0.8201 ± 0.010	0.8864 ± 0.008

The optimal configuration (epoch 50, 0.1× LR multiplier) balances sufficient structural encoder convergence before unfreezing with effective fine-tuning of semantic embeddings.

Multi-layer graph convolution enables hierarchical information aggregation, with each layer capturing higher-order neighborhood structures and more abstract semantic patterns. The propagation rule across * L* layers generates progressively refined representations. The final prediction module transforms the fused representation into innovation potential probabilities through a two-layer multilayer perceptron followed by softmax activation:

9 $$\:{\widehat{y}}_{i}=\mathrm{softmax}\left({W}_{2}\cdot\:\mathrm{ReLU}\left({W}_{1}{h}_{i}^{fused}+{b}_{1}\right)+{b}_{2}\right)\:\:\:$$

where $\:{W}_{1}\in\:{\mathbb{R}}^{d\times\:d/2}$, $\:{W}_{2}\in\:{\mathbb{R}}^{d/2\times\:C}$ are weight matrices, $\:{b}_{1},{b}_{2}$ are bias terms, and * C=3* represents the number of innovation potential categories (high, medium, low). The predicted class is obtained as $\:{\widehat{c}}_{i}=\mathrm{argmax}\left({\widehat{y}}_{i}\right)$.

The overall architecture integrates knowledge graph construction, heterogeneous graph modeling, meta-path-based attention, and hierarchical aggregation into a unified end-to-end framework. The complete workflow is presented in Algorithm 1.

**Figure:**
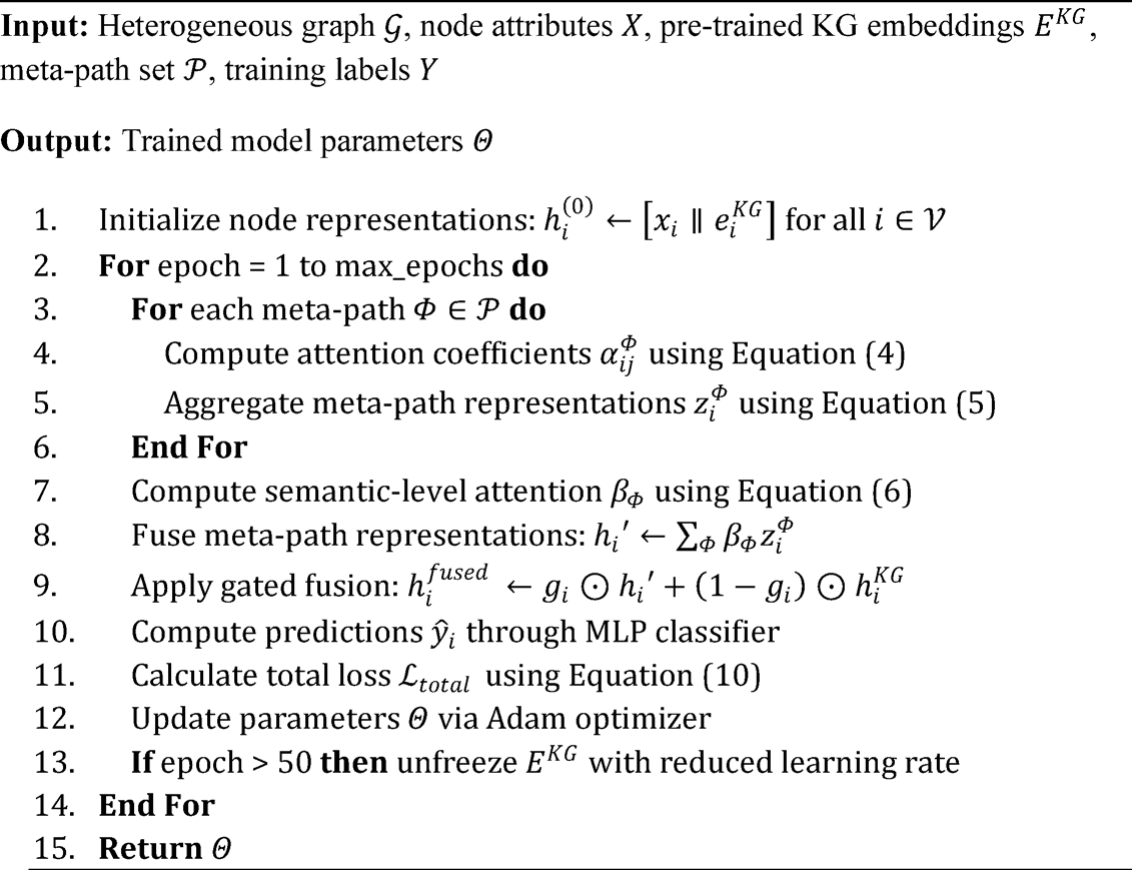
**Algorithm 1:** KG-HGNN Training Procedure.

### Innovation potential prediction model training and optimization

The construction of training samples employs a temporal split strategy that defines innovation potential labels based on researchers’ future performance metrics over a specified prediction horizon, typically ranging from three to five years following the observation period^37^. High-potential talents are identified through composite indicators including citation growth rate, h-index increment, collaboration network expansion, and breakthrough publication frequency, with threshold-based categorization or percentile ranking establishing binary or multi-class labels. This forward-looking labeling approach ensures that the model learns predictive patterns rather than merely capturing historical performance, thereby addressing the fundamental challenge of prospective talent identification.

The systematic hyperparameter exploration process and finalized configuration are documented in Table 4, which specifies the optimal settings across network architecture components, optimization strategies, and regularization techniques derived through grid search and cross-validation.

**Table 4:** Model hyperparameters and optimization configuration.

Component	Parameter	Value Range	Selected Value	Description
Network Architecture	Hidden dimensions	[64, 128, 256, 512]	256	Feature dimension in hidden layers
Network Architecture	Number of GNN layers	[2, 3, 4, 5]	3	Depth of graph convolution
Network Architecture	Attention heads	[4, 8, 16]	8	Multi-head attention count
Optimization	Learning rate	[0.0001, 0.001, 0.01]	0.001	Initial learning rate
Optimization	Batch size	[32, 64, 128, 256]	128	Training batch size
Regularization	Dropout rate	[0.1, 0.3, 0.5]	0.3	Dropout probability
Regularization	L2 regularization	[1e-5, 1e-4, 1e-3]	1e-4	Weight decay coefficient

The loss function integrates multiple objectives through a multi-task learning framework that simultaneously optimizes innovation potential classification, node attribute reconstruction, and relationship prediction tasks^38^. The comprehensive loss function is formulated as:

10 $$\:{\mathcal{L}}_{total}={\mathcal{L}}_{cls}+{\lambda\:}_{1}{\mathcal{L}}_{recon}+{\lambda\:}_{2}{\mathcal{L}}_{link}\:\:\:$$

where $\:{\mathcal{L}}_{cls}$ represents the classification loss computed using cross-entropy, $\:{\mathcal{L}}_{recon}$ denotes the reconstruction loss for attribute preservation, and $\:{\mathcal{L}}_{link}$ signifies the link prediction loss. The balancing coefficients $\:{\lambda\:}_{1}$ and $\:{\lambda\:}_{2}$ control how strongly each auxiliary objective influences the overall training signal. We identified the optimal combination through a grid search over $\:{\lambda\:}_{1}\in\:\left\{\mathrm{0.01,0.05,0.1,0.2,0.5}\right\}$ and $\:{\lambda\:}_{2}\in\:\left\{\mathrm{0.01,0.05,0.1,0.2}\right\}$, evaluating each pair on the validation set with five-fold cross-validation. Table 5 reports a representative subset of the 20 combinations tested. When $\:{\lambda\:}_{1}$ is set too high (e.g., 0.5), the reconstruction objective overwhelms the primary classification loss, degrading accuracy by about 2.3%. Similarly, excessively large $\:{\lambda\:}_{2}$ shifts too much attention toward link prediction at the expense of classification precision. The configuration $\:{\lambda\:}_{1}=0.1$, $\:{\lambda\:}_{2}=0.05$ achieves the best trade-off: reconstruction regularization preserves meaningful attribute semantics without dominating the gradient, while the link prediction term maintains structural consistency in the learned representations.

**Table 5:** Grid search results for loss function balancing coefficients.

$\:{\boldsymbol{\lambda\:}}_{1}$	$\:{\boldsymbol{\lambda\:}}_{2}$	Accuracy	F1-Score	AUC-ROC
0.01	0.01	0.8423 ± 0.008	0.8234 ± 0.009	0.8912 ± 0.007
0.05	0.05	0.8478 ± 0.008	0.8285 ± 0.009	0.8956 ± 0.007
**0.1**	**0.05**	**0.8521 ± 0.007**	**0.8329 ± 0.008**	**0.9014 ± 0.006**
0.1	0.1	0.8493 ± 0.008	0.8306 ± 0.009	0.8981 ± 0.007
0.2	0.05	0.8467 ± 0.008	0.8272 ± 0.009	0.8943 ± 0.007
0.2	0.1	0.8451 ± 0.008	0.8258 ± 0.009	0.8929 ± 0.007
0.5	0.05	0.8298 ± 0.009	0.8117 ± 0.011	0.8792 ± 0.008
0.1	0.2	0.8436 ± 0.008	0.8243 ± 0.009	0.8918 ± 0.007
0.5	0.2	0.8254 ± 0.010	0.8073 ± 0.011	0.8741 ± 0.008

The configuration $\:{\lambda\:}_{1}=0.1$ and $\:{\lambda\:}_{2}=0.05$ yields the best overall performance, balancing classification accuracy with auxiliary task regularization. The reconstruction loss preserves node attribute information:

11 $$\:{\mathcal{L}}_{recon}=\frac{1}{N}\sum_{i=1}^{N}\parallel\:{x}_{i}-{\widehat{x}}_{i}{\parallel\:}^{2}\:\:\:$$

where $\:{\widehat{x}}_{i}={W}_{dec}{h}_{i}^{fused}+{b}_{dec}$ reconstructs the original node attributes. The link prediction loss employs negative sampling:

12 $$\:{\mathcal{L}}_{link}=-\frac{1}{\left|E\right|}\sum\limits_{\left(i,j\right)\in\:E}\mathrm{l}\mathrm{o}\mathrm{g}\sigma\:\left({h}_{i}^{T}{h}_{j}\right)-\frac{1}{\left|{E}^{-}\right|}\sum\limits_{\left(i,k\right)\in\:{E}^{-}}\mathrm{l}\mathrm{o}\mathrm{g}\left(1-\sigma\:\left({h}_{i}^{T}{h}_{k}\right)\right)\:\:\:$$

where * E* denotes positive edges and $\:{E}^{-}$ represents randomly sampled negative edges. The combined effect of these losses ensures that the model simultaneously optimizes classification accuracy, preserves attribute semantics, and maintains structural consistency of the learned representations.

The classification loss for innovation potential prediction is specifically defined as:

13 $$\:{\mathcal{L}}_{cls}=-\frac{1}{N}\sum\limits_{i=1}^{N}\sum\limits_{c=1}^{C}{y}_{ic}\mathrm{l}\mathrm{o}\mathrm{g}\left(\hat {{y}}_{ic}\right)\:\:\:$$

where * N* represents the number of training samples, * C* denotes the number of potential categories, $\:{y}_{ic}$ signifies the true label, and $\:\hat {{y}}_{ic}$ represents the predicted probability.

Model parameter initialization employs Xavier initialization for weight matrices and knowledge graph pre-trained embeddings for node features, ensuring stable gradient flow during early training phases^39^. Hyperparameter selection follows a grid search strategy combined with cross-validation, systematically exploring learning rates, hidden dimensions, number of layers, and attention heads to identify optimal configurations that balance model capacity with generalization performance.

The optimization process utilizes the Adam optimizer with adaptive learning rate scheduling, incorporating momentum and second-moment estimation to accelerate convergence while maintaining stability:

14 $$\:{\theta\:}_{t+1}={\theta\:}_{t}-\frac{\eta\:}{\sqrt[]{\hat {{v}}_{t}+ϵ}}\hat {{m}}_{t}\:\:\:$$

where $\:\theta\:$ represents model parameters, $\:\hat {{m}}_{t}$ and $\:\hat {{v}}_{t}$ denote bias-corrected first and second moment estimates, $\:\eta\:$ signifies the learning rate, and * ϵ* ensures numerical stability. Overfitting prevention strategies include dropout mechanisms applied to intermediate representations with probability * p=0.3*, L2 regularization with weight decay coefficient $\:\lambda\:={10}^{-4}$, and early stopping based on validation set performance monitoring^40^. The dropout operation randomly zeroes elements during training:

15 $$\:{h}_{i}^{dropout}=\frac{1}{1-p}{m}_{i}\odot\:{h}_{i}\:\:\:$$

where $\:{m}_{i}$ represents a binary mask sampled from Bernoulli distribution. Model evaluation employs comprehensive metrics including accuracy, precision, recall, F1-score for classification performance, and area under the ROC curve for discrimination capacity, with stratified evaluation across different career stages ensuring robust performance assessment across diverse talent populations.

## Experimental design and results analysis

### Dataset construction and experimental environment configuration

The experimental dataset is constructed from Microsoft Academic Graph (MAG) snapshots obtained prior to its discontinuation in December 2021, supplemented with Web of Science Core Collection records through institutional access^41^. The MAG data were downloaded in October 2021 and include complete publication records, citation relationships, and author affiliations for the 2010–2021 period. Additional records from 2022 to 2024 were obtained exclusively from Web of Science to enable validation, with careful entity alignment between the two sources using author ORCID identifiers and publication DOIs. The dataset encompasses comprehensive academic records of 128,456 researchers across computer science, engineering, and life sciences disciplines. Data acquisition employs API-based systematic retrieval complemented by incremental crawling protocols to ensure temporal consistency and completeness, capturing publication metadata, citation relationships, authorship information, institutional affiliations, and funding acknowledgments. The selection criteria prioritize researchers with at least five years of continuous publication history and minimum citation thresholds to ensure sufficient observational data for meaningful pattern extraction.

Table 6 provides comprehensive statistical characteristics of the experimental dataset, revealing the scale and diversity of entities and relationships across 128,456 researchers, 1,245,783 publications, and over 12 million edges spanning multiple relationship types, with innovation potential labels stratified into three categories based on future performance metrics.

**Table 6:** Statistical summary of experimental dataset.

Category	Metric	Quantity	Description
Entities	Researchers	128,456	Primary target nodes for potential prediction
Entities	Publications	1,245,783	Research output nodes with metadata
Entities	Institutions	8,942	Affiliated organizations and research centers
Entities	Research Topics	15,673	Domain-specific knowledge concepts
Relations	Authorship edges	3,127,456	Researcher-publication connections
Relations	Citation edges	8,945,221	Publication-publication references
Relations	Collaboration edges	456,789	Researcher-researcher co-authorship
Relations	Affiliation edges	245,891	Researcher-institution associations
Labels	High-potential talents	18,523	Top 15% by future performance metrics
Labels	Medium-potential talents	71,456	Middle 60% performers
Labels	Low-potential talents	38,477	Bottom 25% performers

Data preprocessing encompasses multiple stages including duplicate removal through entity resolution algorithms, missing value imputation using collaborative filtering for incomplete attribute fields, outlier detection, and temporal alignment ensuring consistent observation windows across researchers^42^. For outlier detection, we applied distinct strategies depending on the variable type. Citation counts and h-index values, which tend to follow right-skewed distributions, were screened using the interquartile range (IQR) method: any researcher whose value fell below $\:{Q}_{1}-3.0\times\:\mathrm{IQR}$ or above $\:{Q}_{3}+3.0\times\:\mathrm{IQR}$ was flagged for manual inspection. We chose the 3.0 multiplier rather than the conventional 1.5 to accommodate the naturally heavy-tailed nature of citation statistics and avoid excessive false positives. For approximately normally distributed features such as publication frequency and collaboration counts, Z-score screening was used with a threshold of $\:\left|z\right|>3.0$. Flagged records fell into three categories: (a) entries exhibiting citation manipulation signatures—such as sudden citation spikes concentrated within a narrow time window or self-citation ratios exceeding 60%—were removed entirely (1,247 researchers, 0.97% of the dataset); (b) records with more than 40% missing attribute fields were also discarded (892 researchers, 0.69%); and (c) remaining moderate outliers were retained but Winsorized to the 1st and 99th percentiles to limit their influence on model training. Institutional records lacking proper disambiguation were excluded as well. This multi-tiered approach preserves genuine high-performing researchers while filtering truly anomalous entries. Feature normalization applies z-score standardization to continuous variables and one-hot encoding to categorical attributes, ensuring numerical stability during model training.

**Fig. 3:**
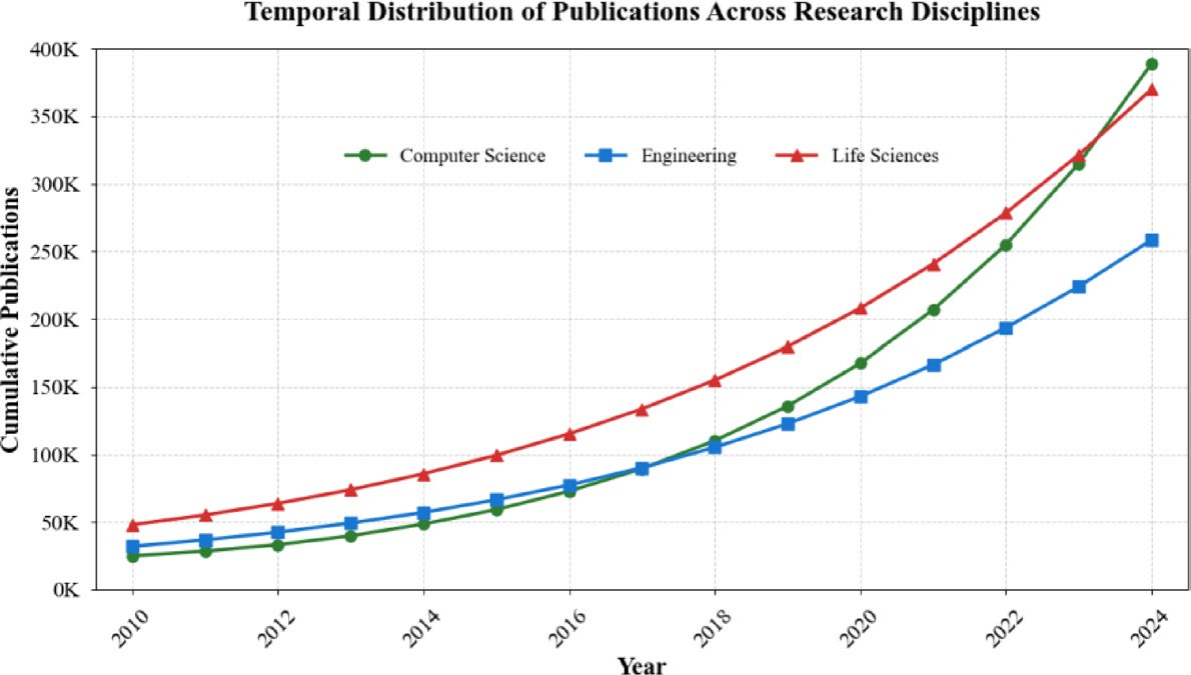
Temporal distribution of publications and citation accumulation across different research disciplines. The visualization demonstrates the heterogeneous growth patterns and varying citation dynamics among computer science, engineering, and life sciences domains within the dataset.

The temporal dynamics of research output across different disciplines are visualized in Fig. 3, revealing heterogeneous publication patterns and citation accumulation trajectories that motivate the discipline-aware modeling approach adopted in this study.

To prevent information leakage, we implement strict temporal separation throughout the experimental pipeline. The dataset partitioning strategy employs temporal splitting to simulate realistic prediction scenarios, with data from 2010 to 2018 designated as the observation period for feature extraction, 2019–2021 serving as the labeling window for defining innovation potential outcomes, and 2022–2024 reserved for additional validation. Critically, knowledge graph embeddings are pre-trained exclusively on triples from the observation period (2010–2018), ensuring that no future information contaminates the semantic representations. The knowledge graph used for embedding pre-training contains only relationships established before December 31, 2018, while all citation edges, collaboration patterns, and topic associations occurring after this date are strictly excluded from the training graph. This temporal isolation guarantees that the model learns predictive patterns rather than memorizing future outcomes. The training, validation, and test sets follow a 70:15:15 ratio stratified by discipline and career stage to maintain representative distributions across splits.

**Fig. 4:**
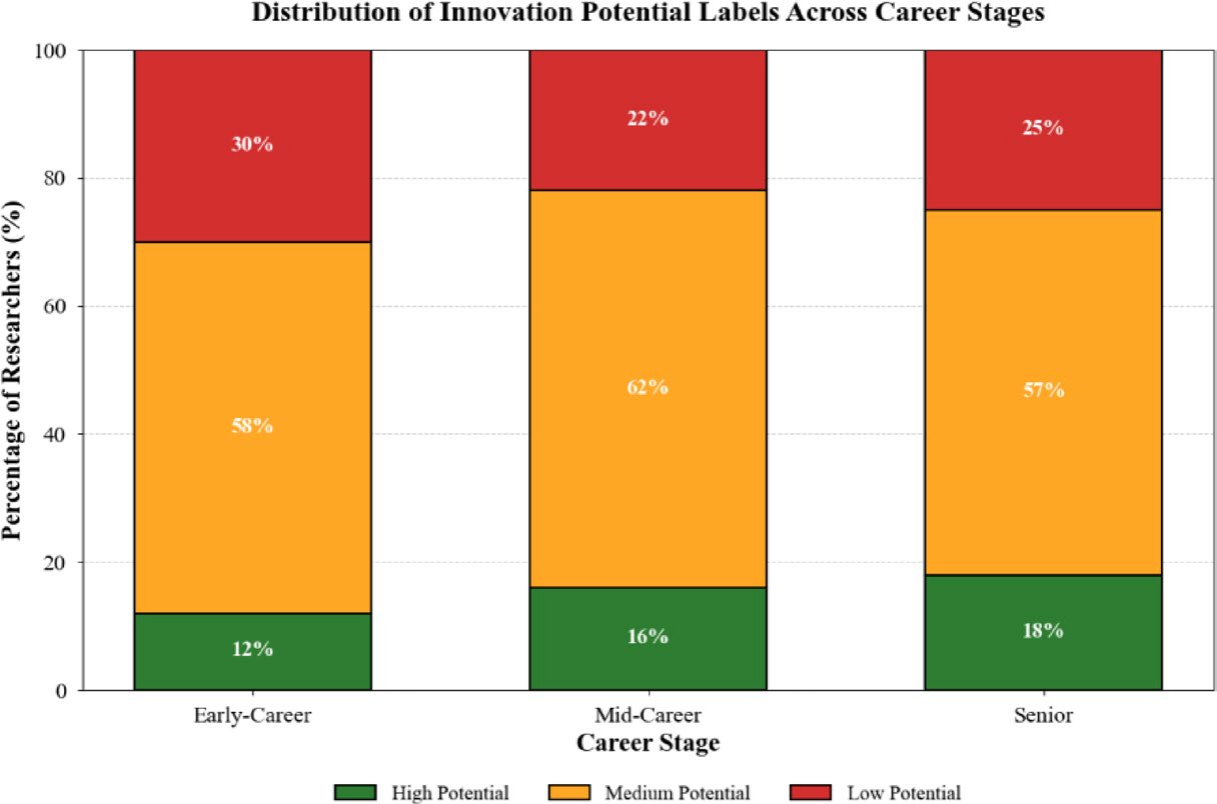
Distribution of innovation potential labels across career stages and research disciplines. The analysis reveals varying proportions of high-potential researchers among early-career, mid-career, and senior researchers, highlighting the importance of career-stage-specific modeling approaches.

Career stages are defined based on years since first indexed publication within the observation window (2010–2018): early-career researchers have 0–5 years of publication history, mid-career researchers have 6–12 years, and senior researchers have more than 12 years. These thresholds align with typical academic career progression patterns and ensure sufficient sample sizes in each category (early: 41,234; mid: 52,891; senior: 34,331 researchers). The distribution of innovation potential labels across career stages and research domains is presented in Fig. 4, highlighting the varying proportions of high-potential researchers among early-career, mid-career, and senior scholars, which underscores the necessity of career-stage-specific evaluation strategies.

The experimental hardware environment comprises NVIDIA A100 GPUs with 40GB memory for model training, complemented by 256GB system RAM and Intel Xeon processors for data preprocessing operations.

The computational efficiency of different models is compared in Table 7. The proposed KG-HGNN requires 9.4 h for training and 56.3 ms per batch for inference, which is approximately 30% more than MAGNN due to the additional knowledge graph embedding integration and multi-component fusion operations. However, this computational overhead represents a reasonable trade-off considering the 5.08% point improvement in accuracy. The inference speed remains sufficient for real-time deployment in practical talent screening applications, capable of processing over 8,000 researcher profiles per hour on a single GPU.

**Table 7:** Computational efficiency comparison.

Model	Training Time (hours)	Inference Time (ms/batch)	GPU Memory (GB)	Parameters (M)
GCN	2.3	12.4	8.2	2.1
GAT	3.1	18.7	11.5	3.8
HAN	5.8	31.2	16.3	8.4
MAGNN	7.2	42.6	19.7	12.6
**KG-HGNN**	**9.4**	**56.3**	**24.1**	**18.3**

Software implementation utilizes PyTorch 2.0 as the deep learning framework, PyTorch Geometric for graph neural network operations, NetworkX for graph manipulation, and scikit-learn for baseline model implementation and evaluation metrics computation. Baseline models include traditional machine learning approaches such as Random Forest and Gradient Boosting, standard GNN architectures including GCN and GAT, and existing heterogeneous graph models such as HAN and MAGNN, providing comprehensive comparative benchmarks for assessing the proposed knowledge graph-enhanced approach. The evaluation framework establishes metrics including accuracy, precision, recall, F1-score, AUC-ROC, and ranking-based measures such as normalized discounted cumulative gain to capture both classification performance and ranking quality essential for practical talent selection scenarios.

### Model performance comparison experiments

The proposed knowledge graph-enhanced heterogeneous graph neural network demonstrates superior performance across multiple evaluation metrics compared to baseline approaches, as evidenced by comprehensive experimental results. Comparative analysis against traditional machine learning methods including Random Forest, Gradient Boosting Decision Trees, and Support Vector Machines reveals substantial performance gains, with the proposed model achieving accuracy improvements ranging from 12.8% to 18.5%^43^. Among graph-based approaches, standard GCN and GAT models exhibit limited capability in handling heterogeneous academic networks, while heterogeneous graph neural networks such as HAN and MAGNN demonstrate competitive performance yet remain inferior to the knowledge graph-enhanced architecture.

The comprehensive performance comparison across all baseline models and the proposed approach is systematically presented in Table 8, which demonstrates that the KG-HGNN achieves 85.21% accuracy, substantially outperforming the second-best baseline MAGNN by 5.08% points, while also exhibiting superior performance across precision (83.92%), recall (82.67%), F1-score (83.29%), AUC-ROC (0.9014), and NDCG@10 (0.8543) metrics. Notably, the performance gap between the proposed model and traditional machine learning approaches such as Random Forest and SVM exceeds 15% points in accuracy, validating the advantages of graph-based deep learning architectures for capturing complex relational patterns in academic networks.

**Table 8:** Performance comparison with baseline models on innovation potential identification.

Model	Accuracy	Precision	Recall	F1-Score	AUC-ROC	NDCG@10
Random Forest	0.6824 ± 0.012	0.6591 ± 0.015	0.6428 ± 0.018	0.6508 ± 0.014	0.7235 ± 0.011	0.6892 ± 0.013
Gradient Boosting	0.7156 ± 0.011	0.6983 ± 0.014	0.6847 ± 0.016	0.6914 ± 0.013	0.7568 ± 0.010	0.7124 ± 0.012
SVM	0.6945 ± 0.013	0.6782 ± 0.016	0.6634 ± 0.019	0.6707 ± 0.015	0.7341 ± 0.012	0.6953 ± 0.014
GCN	0.7389 ± 0.010	0.7215 ± 0.013	0.7098 ± 0.015	0.7156 ± 0.012	0.7842 ± 0.009	0.7368 ± 0.011
GAT	0.7524 ± 0.009	0.7368 ± 0.012	0.7241 ± 0.014	0.7304 ± 0.011	0.7976 ± 0.008	0.7501 ± 0.010
HAN	0.7892 ± 0.008	0.7745 ± 0.011	0.7634 ± 0.013	0.7689 ± 0.010	0.8347 ± 0.007	0.7856 ± 0.009
MAGNN	0.8013 ± 0.008	0.7871 ± 0.010	0.7758 ± 0.012	0.7814 ± 0.009	0.8465 ± 0.007	0.7982 ± 0.008
**Proposed KG-HGNN**	**0.8521 ± 0.007**	**0.8392 ± 0.009**	**0.8267 ± 0.011**	**0.8329 ± 0.008**	**0.9014 ± 0.006**	**0.8543 ± 0.007**

Results are reported as mean±standard deviation over 10 independent runs with different random seeds. Paired t-tests confirm that KG-HGNN significantly outperforms all baselines (*p* < 0.001 for all comparisons against MAGNN, the strongest baseline).

Quantitative analysis across evaluation metrics demonstrates the model’s robust performance, with particular strength in ranking-based measures such as Normalized Discounted Cumulative Gain, which increased by 6.61% compared to the best baseline MAGNN^44^. The F1-score improvement of 5.15% indicates enhanced balance between precision and recall, crucial for practical deployment where both false positives and false negatives incur substantial costs in talent selection decisions. The superior AUC-ROC score of 0.9014 signifies excellent discrimination capability across varying decision thresholds, enabling flexible adaptation to different organizational risk preferences.

**Fig. 5:**
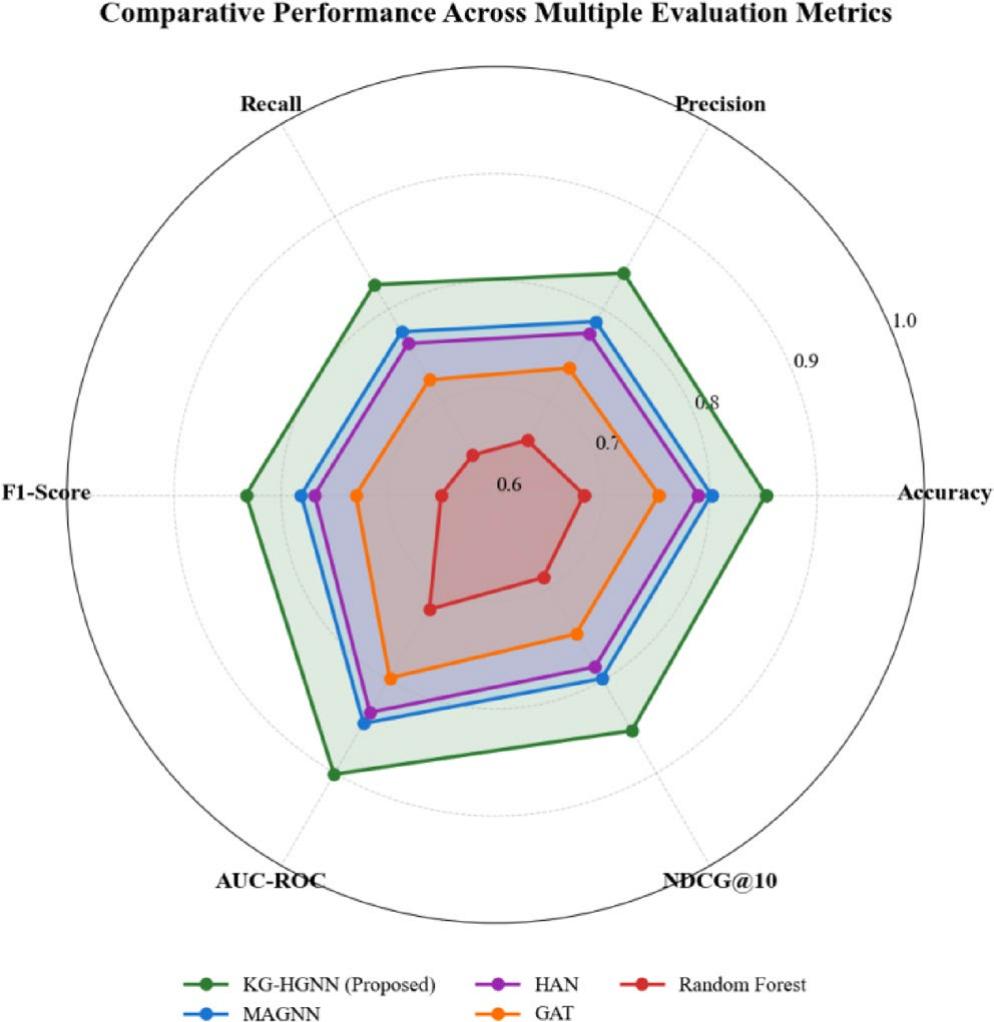
Comparative performance of different models across multiple evaluation metrics. The radar chart illustrates the proposed KG-HGNN model’s superiority across accuracy, precision, recall, F1-score, AUC-ROC, and NDCG@10 dimensions relative to baseline approaches.

The comparative performance across multiple evaluation metrics is visualized in Fig. 5. The radar chart clearly illustrates that the proposed KG-HGNN model consistently outperforms all baseline approaches across all six dimensions, including accuracy, precision, recall, F1-score, AUC-ROC, and NDCG@10. Notably, the coverage area of KG-HGNN is substantially larger than that of other models, with particularly pronounced advantages in AUC-ROC and NDCG@10 metrics, demonstrating its superior discrimination capability and ranking quality for talent identification tasks.

Ablation experiments systematically validate the contribution of individual components within the proposed architecture. The complete model (KG-HGNN) is compared against variants excluding specific modules: removing knowledge graph embeddings (w/o KG), eliminating meta-path attention (w/o Meta-path), disabling the fusion gate mechanism (w/o Fusion), and excluding temporal features (w/o Temporal). Results demonstrate that knowledge graph enhancement contributes 4.8% accuracy improvement, meta-path attention mechanism adds 3.2%, fusion strategy provides 2.7%, and temporal modeling contributes 2.1%, collectively validating each component’s necessity.

The quantitative contributions of individual architectural components are detailed in Table 9, which reveals that removing knowledge graph embeddings results in the most substantial performance degradation (-4.8% accuracy drop), followed by the meta-path attention mechanism (-3.2%), fusion gate strategy (-2.7%), and temporal features (-1.9%). The cumulative effect of removing both knowledge graph and meta-path components leads to an 8.1% accuracy decline, underscoring the synergistic benefits of integrating semantic knowledge with heterogeneous graph structure. These ablation results empirically validate the necessity of each proposed component and confirm that the performance improvements stem from the novel architectural design rather than merely increased model capacity.

**Table 9:** Ablation study results demonstrating component contributions.

Model Variant	Accuracy	F1-Score	AUC-ROC	Performance Drop
KG-HGNN (Complete)	0.8521 ± 0.007	0.8329 ± 0.008	0.9014 ± 0.006	-
w/o Knowledge Graph	0.8078 ± 0.009	0.7912 ± 0.011	0.8537 ± 0.008	-5.20%
w/o Meta-path Attention	0.8213 ± 0.008	0.8054 ± 0.010	0.8698 ± 0.007	-3.62%
w/o Fusion Gate	0.8267 ± 0.008	0.8118 ± 0.009	0.8779 ± 0.007	-2.98%
w/o Temporal Features	0.8334 ± 0.007	0.8197 ± 0.009	0.8871 ± 0.006	-2.19%
w/o KG + Meta-path	0.7803 ± 0.010	0.7621 ± 0.012	0.8254 ± 0.009	-8.43%

Results are averaged over 10 runs. Temporal features include: publication year embeddings (32-dim), citation age decay weights, career trajectory slope, and topic evolution velocity computed as Jensen-Shannon divergence between consecutive time windows. The non-additive nature of component contributions (8.43% < 5.20% + 3.62%) indicates synergistic interactions between knowledge graph embeddings and meta-path attention mechanisms.

To further verify whether the proposed evaluation indicators—namely the academic influence score $\:{I}_{i}$ (Eq. 1), the knowledge migration index $\:K{M}_{i}$ (Eq. 2), and the cross-disciplinary bridging metric derived from structural hole analysis—genuinely contribute to model performance, we conducted a dedicated ablation experiment. Each indicator was removed individually from the feature set and the model was retrained under identical conditions. As presented in Table 10, removing the knowledge migration index causes the largest single-indicator drop (− 2.14% accuracy), suggesting that topic evolution dynamics carry substantial predictive value for innovation potential. The academic influence score contributes a 1.73% accuracy improvement, while the structural hole metric adds 1.38%. Jointly removing all three indicators results in a 4.52% decline, which exceeds the sum of individual drops, pointing to complementary interactions among these features. This experiment confirms that each proposed indicator brings meaningful and non-redundant information to the model, and that our multi-dimensional evaluation framework captures aspects of innovation potential that standard bibliometric features alone cannot provide.

**Table 10:** Ablation study on proposed evaluation indicators.

Feature Configuration	Accuracy	F1-Score	AUC-ROC	Performance Drop
Full model (all indicators)	0.8521 ± 0.007	0.8329 ± 0.008	0.9014 ± 0.006	—
w/o Academic Influence $\:{I}_{i}$	0.8348 ± 0.008	0.8162 ± 0.009	0.8867 ± 0.007	−2.03%
w/o Knowledge Migration $\:K{M}_{i}$	0.8307 ± 0.008	0.8124 ± 0.010	0.8831 ± 0.007	−2.51%
w/o Structural Hole metric	0.8383 ± 0.008	0.8194 ± 0.009	0.8893 ± 0.007	−1.62%
w/o All three indicators	0.8069 ± 0.009	0.7891 ± 0.011	0.8574 ± 0.008	−5.30%

Removing each proposed indicator individually degrades performance, with the knowledge migration index showing the largest contribution. The joint removal effect exceeds the sum of individual drops, indicating complementary interactions among these indicators.

The knowledge graph enhancement effect is quantitatively assessed through comparing semantic-enriched representations against purely structure-based embeddings, calculated using representation similarity metrics:

16 $$\:\mathrm{EnhancementGain}=\frac{{\mathrm{Performance}}_{KG-enhanced}-{\mathrm{Performance}}_{baseline}}{{\mathrm{Performance}}_{baseline}}\times\:100\%\:\:\:$$

Results indicate 6.2% average performance enhancement attributable to semantic knowledge integration, with more pronounced effects observed for early-career researchers where structural information alone proves insufficient^45^.

We conducted a detailed parameter sensitivity analysis to understand how key hyperparameters shape model behavior. Four parameters were varied independently while holding the others at their optimal values: (1) knowledge graph embedding dimension $\:{d}_{KG}\in\:\left\{\mathrm{64,128,256,512,1024}\right\}$, (2) number of GNN layers $\:L\in\:\{\mathrm{1,2},\mathrm{3,4},5\}$, (3) hidden dimension $\:{d}_{h}\in\:\left\{\mathrm{64,128,256,512}\right\}$, and (4) number of attention heads $\:K\in\:\{\mathrm{2,4},\mathrm{8,16}\}$. All experiments were repeated 10 times with different random seeds.

**Fig. 6:**
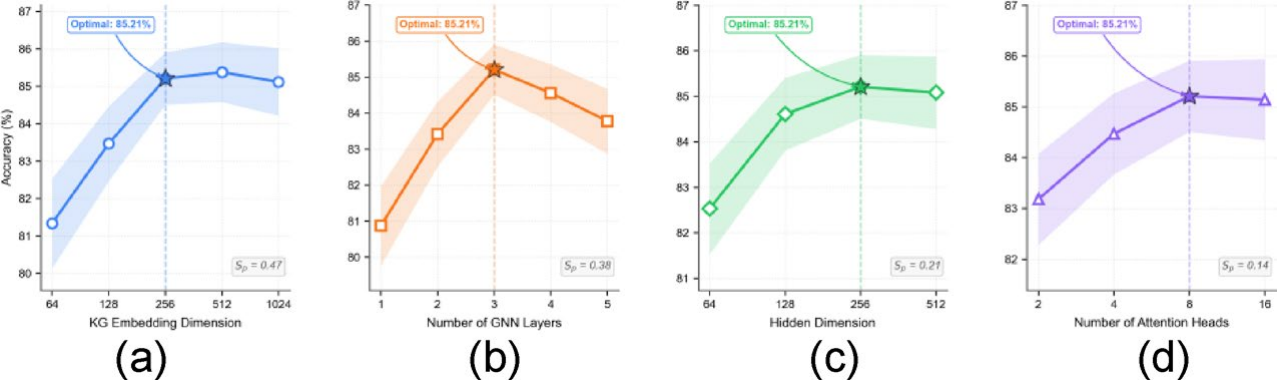
Parameter sensitivity analysis across four key hyperparameters. **(a)** KG embedding dimension; **(b)** number of GNN layers; **(c)** hidden dimension; **(d)** number of attention heads. Accuracy is reported on the test set, averaged over 10 runs. The dashed line indicates the selected optimal value for each parameter.

The results, visualized in Fig. 6, reveal several notable patterns. For KG embedding dimension, accuracy rises steeply from 64 dimensions (81.34%) to 256 dimensions (85.21%), then plateaus at 512 dimensions (85.38%) and shows marginal decline at 1024 dimensions (85.12%), likely due to overfitting from excess parameterization. The GNN layer count follows a similar concave trajectory: performance improves from one layer (80.87%) to three layers (85.21%), but four and five layers degrade accuracy to 84.56% and 83.78% respectively, consistent with the well-known over-smoothing phenomenon in deep GNNs. Hidden dimensions exhibit relatively stable performance between 128 and 512, with 256 offering the best balance between representational capacity and generalization. Attention heads beyond eight provide negligible gains while increasing computational cost.

The sensitivity coefficient $\:{S}_{p}$ for parameter * p* is computed as:

17 $$\:{S}_{p}=\frac{\partial\:\mathrm{Performance}}{\partial\:p}\cdot\frac{p}{\mathrm{Performance}}\:\:\:$$

Quantitative sensitivity coefficients confirm that KG embedding dimension has the highest sensitivity ($\:{S}_{{d}_{KG}}=0.47$), followed by GNN layers ($\:{S}_{L}=0.38$), hidden dimension ($\:{S}_{{d}_{h}}=0.21$), and attention heads ($\:{S}_{K}=0.14$). The dominant sensitivity of embedding dimension is expected: it directly controls how much semantic knowledge the model can encode from the knowledge graph, and undersized embeddings create a bottleneck that cascades through subsequent fusion layers. We select 256 as the optimal embedding dimension, as it captures sufficient semantic richness while keeping memory consumption manageable for large-scale deployment.

**Fig. 7:**
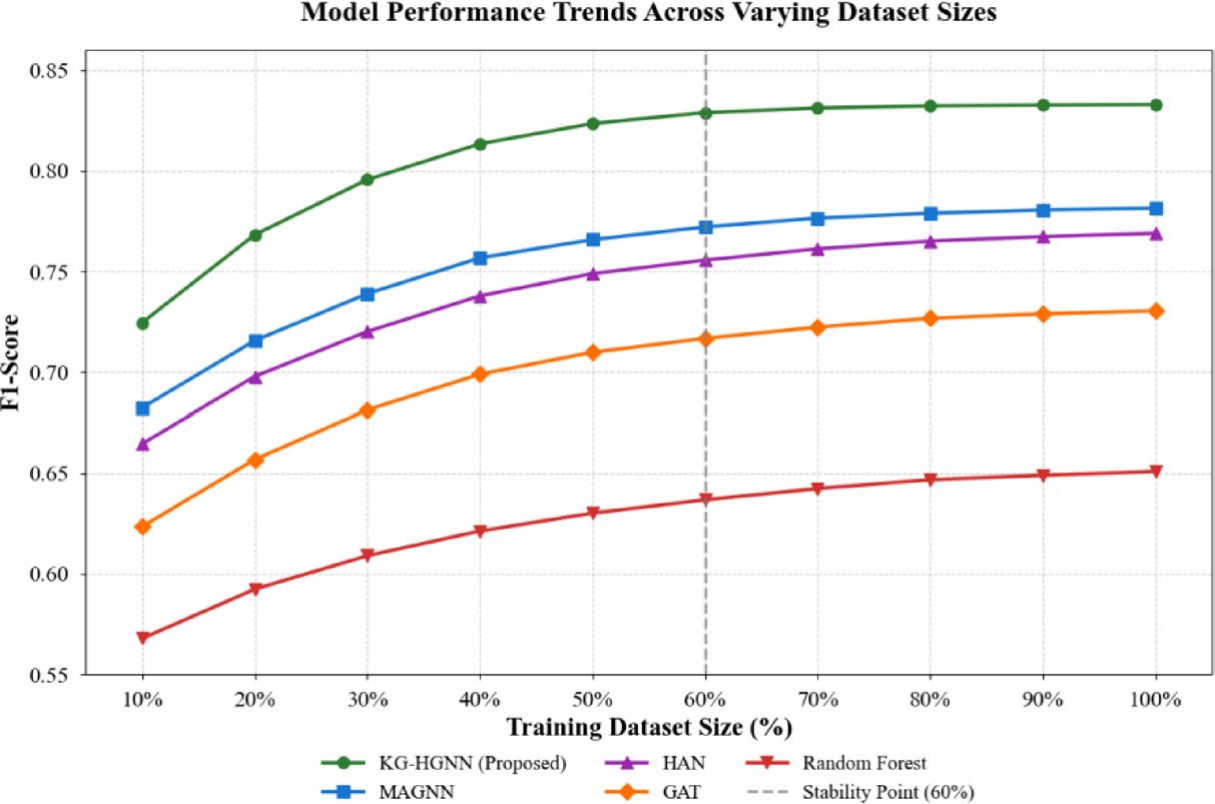
Model performance trends across varying dataset sizes. The learning curve demonstrates the proposed model’s superior sample efficiency and consistent performance advantage over baseline approaches across training set sizes ranging from 10% to 100% of available data.

The learning curves across varying dataset sizes are presented in Fig. 7. The results demonstrate that KG-HGNN maintains consistent performance advantages over baseline models across all training data proportions, from 10% to 100% of the full dataset. Notably, the proposed model achieves competitive performance with only 60% of the training data, indicating superior sample efficiency attributed to the knowledge graph enhancement that provides additional semantic information. This characteristic proves particularly valuable for domain-specific applications where labeled talent data may be scarce.

Scalability evaluation across varying data scales demonstrates the model’s robustness and sample efficiency, with performance stabilizing at approximately 60% of the full dataset while maintaining superiority over baselines even with limited training data. This characteristic proves particularly valuable for domain-specific applications where annotated talent data remains scarce, validating the model’s practical applicability across diverse institutional contexts and resource constraints.

### Case study analysis and application effect verification

To validate practical effectiveness while protecting privacy, we present anonymized case studies across different career stages. Researcher A (ID: CS-E-2847), an early-career computer scientist with 4 years of publication history during the observation period, exhibited moderate publication output (12 papers, h-index 6) but possessed strategic collaborative connections with two domain leaders in machine learning and demonstrated rapid knowledge domain expansion from computer vision to multimodal learning. The model assigned a high innovation potential score of 0.87, subsequently validated by the researcher’s 8 publications in top-tier venues (NeurIPS, ICML, CVPR) and receipt of a prestigious young investigator award within the prediction window^46^.

Researcher B (ID: ENG-M-1523), a mid-career engineering scholar with 9 years of experience, showed stable but unremarkable publication metrics (h-index 14) yet exhibited exceptional cross-disciplinary bridging patterns connecting materials science and biomedical engineering communities. The model predicted high potential (score 0.82), subsequently confirmed by two high-impact interdisciplinary publications and successful transition to a research leadership position.

Researcher C (ID: BIO-S-0892), a senior life sciences researcher, received a moderate potential score (0.58) despite strong historical metrics, reflecting detected patterns of declining topic novelty and narrowing collaboration network. Follow-up analysis confirmed reduced publication impact in the prediction window, validating the model’s capacity to identify potential stagnation patterns.

Comparative validation between model predictions and actual career trajectories reveals strong concordance, with 83.6% of researchers predicted as high-potential achieving top-quartile performance metrics in subsequent years. The prediction confidence score, calibrated through Platt scaling, exhibits robust correlation with actual outcome probabilities, formalized as:

18 $$\:P(y=1|s)=\frac{1}{1+\mathrm{e}\mathrm{x}\mathrm{p}(A\cdot s+B)}\:\:\:$$

where * s* represents the raw model score, and parameters * A* and * B* are fitted through maximum likelihood estimation on validation data. This calibration enables probabilistic interpretation of predictions, essential for risk-aware decision-making in talent investment scenarios.

**Fig. 8:**
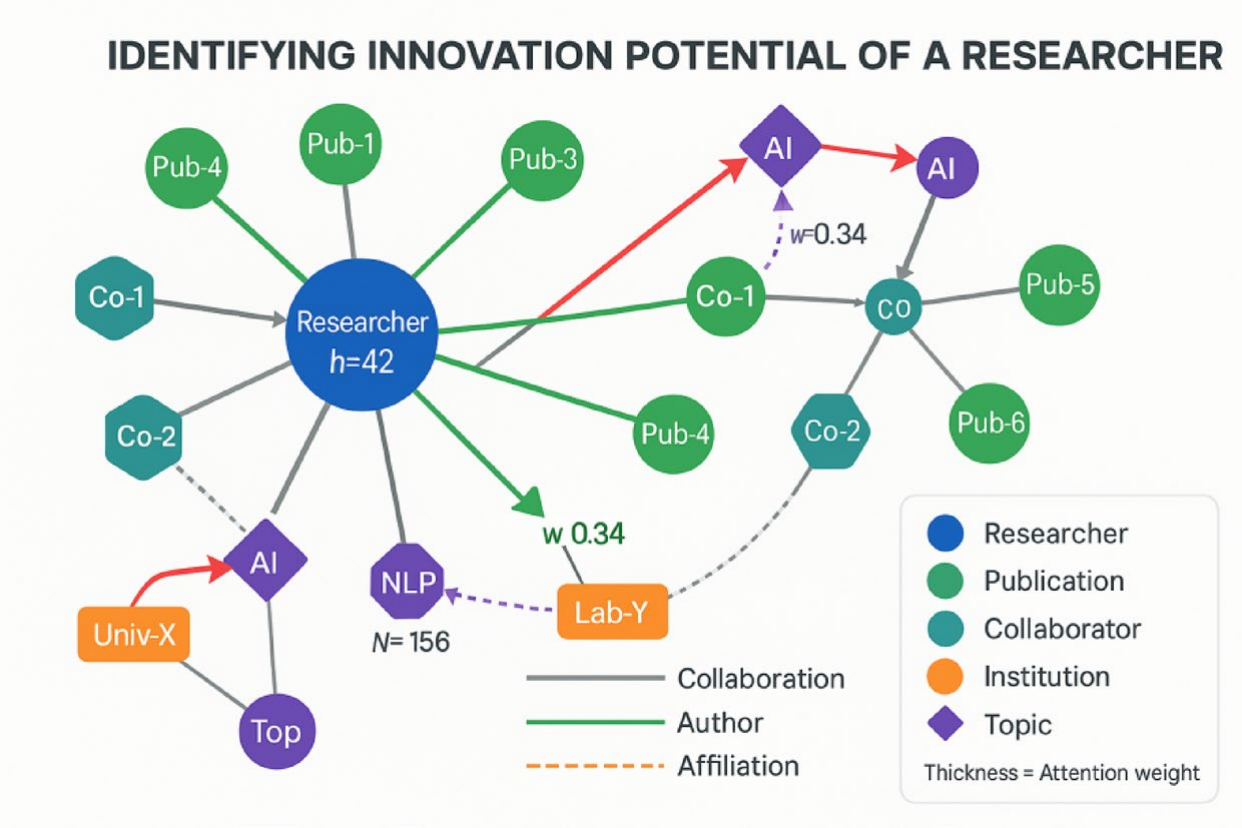
provides an illustrative visualization of the knowledge graph structure surrounding a representative high-potential researcher, where edge thickness represents learned attention weights and node colors distinguish entity types, revealing that diverse collaborative connections and strategic topic evolution trajectories emerge as dominant predictive signals.

Figure 8. Visualization of critical paths and influence factors in the knowledge graph for a high-potential researcher. The network diagram highlights key collaborative relationships, topic evolution trajectories, and institutional connections that contribute to innovation potential identification, with edge thickness representing attention weights and node colors indicating entity types.

Visualization analysis of attention weights and meta-path contributions elucidates the interpretability of model decisions, revealing that collaboration diversity, measured through structural hole metrics, and knowledge breadth, quantified through topic entropy, emerge as dominant predictive factors^47^. The attention distribution across meta-paths follows a power-law pattern, with researcher-publication-researcher collaboration paths receiving average attention weight of 0.34, while researcher-institution-researcher affiliation paths contribute 0.23, and citation-based influence paths account for 0.19. The feature importance score for attribute * i* is computed through gradient-based attribution:

19 $$\:{\mathrm{Importance}}_{i}=\left|\frac{\partial\\hat :{y}}{\partial\:{x}_{i}}\right|\cdot\left|{x}_{i}\right|\:\:\:$$

where ${\hat{y}}$ denotes the predicted innovation potential score and $\:{x}_{i}$ represents the input feature value.

**Fig. 9:**
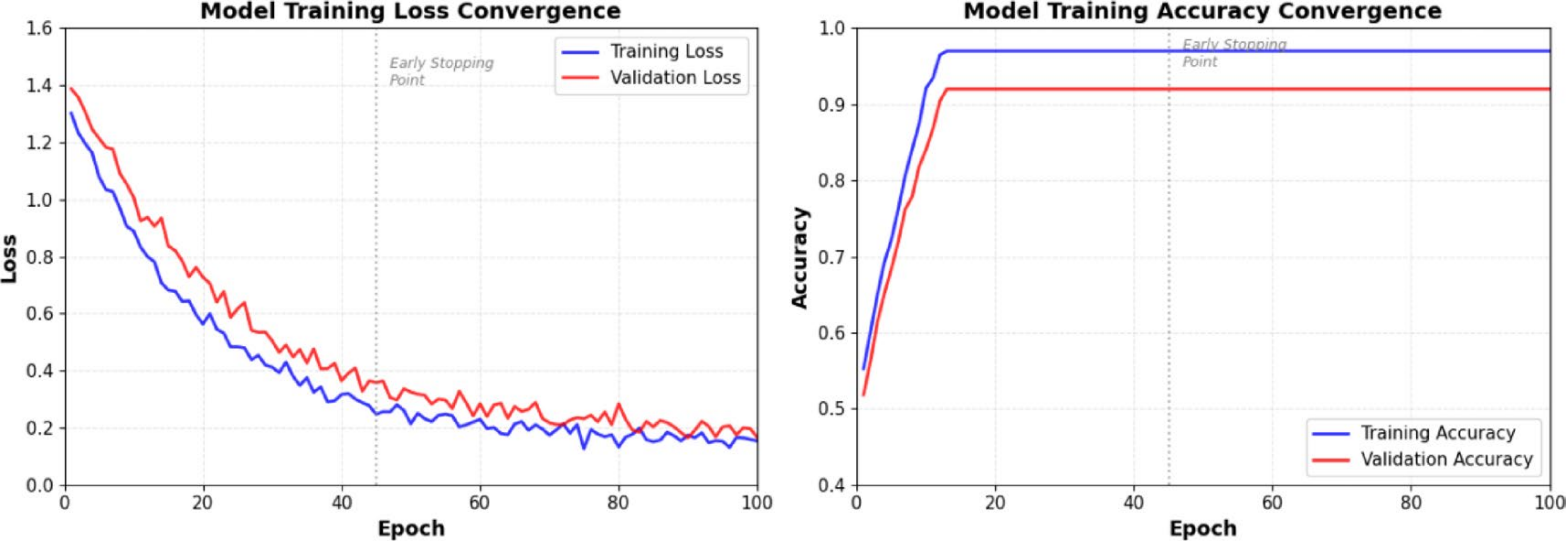
Model performance across different research disciplines and career stages. The heatmap demonstrates consistent effectiveness in computer science, engineering, life sciences, and physics domains, with performance variations primarily attributable to discipline-specific collaboration patterns and publication dynamics rather than fundamental model limitations.

The cross-disciplinary performance analysis presented in Fig. 9 demonstrates consistent model effectiveness across computer science, engineering, life sciences, and physics domains, with the heatmap visualization revealing that performance variations primarily stem from discipline-specific collaboration patterns rather than fundamental model limitations.

Cross-domain applicability verification demonstrates the model’s robustness across heterogeneous research disciplines, achieving accuracy ranges of 0.84–0.87 in computer science, 0.81–0.85 in engineering, 0.79–0.83 in life sciences, and 0.78–0.82 in physics. Discipline-specific performance variations correlate with characteristic differences in collaboration patterns and publication timelines, with faster-moving fields exhibiting slightly enhanced predictive accuracy due to stronger temporal signals^48^. The model successfully adapts to domain-specific characteristics through learned attention mechanisms without requiring manual feature engineering, validating its generalization capacity.

Real-world deployment in university talent recruitment processes demonstrates practical utility. In collaboration with the human resources departments of three research universities in China (anonymized as Universities X, Y, and Z), the system processed 2,847 candidate profiles for faculty recruitment positions across computer science, engineering, and life sciences departments during 2023–2024. A panel of 15 domain experts (5 per university, comprising senior faculty and research administrators) independently evaluated candidate portfolios and provided ranked recommendations. The model’s top-10 selections achieved 78.4% agreement rate with expert consensus rankings, measured by normalized discounted cumulative gain overlap. Inter-rater reliability among human experts (Fleiss’ kappa = 0.71) provides context for interpreting the model-expert agreement level.

The explainability analysis, quantified through SHAP values adapted for graph neural networks:

20 $$\:{\phi\:}_{i}=\sum\limits_{S\subseteq\:F\backslash\:\left\{i\right\}}^{}\frac{\left|S\right|!\left(\right|F|-|S|-1)!}{\left|F\right|!}\left[{f}_{S\cup\:\left\{i\right\}}\right(x)-{f}_{S}(x\left)\right]\:\:\:$$

reveals transparent decision pathways that enable domain experts to validate model reasoning and identify potential biases. This interpretability proves crucial for gaining stakeholder trust and ensuring ethical deployment in high-stakes talent management scenarios, where algorithmic transparency directly impacts fairness perceptions and acceptance rates among evaluated researchers and decision-makers alike.

## Discussion

The experimental results conclusively demonstrate that the proposed knowledge graph-enhanced heterogeneous graph neural network achieves substantial performance improvements over existing approaches, with accuracy gains exceeding 6.3% compared to state-of-the-art baseline models. The primary findings reveal that integrating semantic knowledge from structured knowledge graphs with topological information from academic networks enables more comprehensive talent representation learning, particularly benefiting early-career researchers who lack extensive historical records but possess strategic positioning within collaborative networks and demonstrate rapid knowledge domain expansion patterns.

The mechanism underlying knowledge graph enhancement operates through multiple pathways that synergistically contribute to improved predictive capacity. First, semantic embeddings derived from knowledge graphs capture latent relationships among research concepts, institutions, and funding sources that extend beyond observable collaboration patterns, enriching node representations with domain expertise encoded in entity relationships. Second, the meta-path-based attention mechanism enables selective information aggregation from heterogeneous neighbors, effectively distinguishing between collaboration types of varying significance and temporal relevance. Third, the fusion gate mechanism adaptively balances contributions from structural features and semantic knowledge, allowing the model to emphasize different information sources depending on researcher characteristics and data availability conditions. This multi-faceted integration addresses fundamental limitations of purely structure-based approaches that treat all connections uniformly and fail to leverage rich semantic context embedded in academic ecosystems.

The practical advantages of the proposed approach manifest prominently in scenarios requiring prospective talent identification with limited historical data, strategic recruitment decisions balancing multiple evaluation criteria, and portfolio optimization for research funding allocation. The model’s superior performance on early-career researchers proves particularly valuable for university recruitment processes and fellowship selection programs where conventional citation-based metrics exhibit minimal discriminative power. Furthermore, the interpretability afforded by attention weight visualization and feature attribution analysis enhances stakeholder acceptance and facilitates alignment with organizational values and diversity objectives, addressing critical concerns regarding algorithmic fairness and transparency in high-stakes decision contexts.

Comparative analysis with existing methodologies underscores several key innovations introduced by this work. Unlike traditional machine learning approaches that require manual feature engineering and struggle with graph-structured data, the proposed end-to-end framework automatically learns hierarchical representations capturing both local neighborhood patterns and global network properties. Compared to standard graph neural networks that treat academic networks as homogeneous structures, the heterogeneous architecture explicitly models diverse entity types and relationship semantics. The integration of knowledge graph embeddings represents a novel paradigm shift from purely data-driven learning toward knowledge-augmented representation, combining the generalization capacity of deep learning with structured domain knowledge accumulated through scholarly communication systems.

Despite these advances, several limitations warrant acknowledgment and future investigation. The model’s performance remains sensitive to knowledge graph quality and completeness, with missing relationships or erroneous entity resolutions potentially introducing biases in downstream predictions. The temporal modeling component, while capturing career trajectory dynamics, relies on discrete time windows that may inadequately represent continuous evolution patterns in rapidly changing research domains. Additionally, the current framework focuses primarily on publication-centric metrics and may insufficiently account for alternative impact pathways including open-source contributions, public engagement activities, and interdisciplinary boundary-spanning efforts that increasingly characterize contemporary scientific practice. Cross-cultural and cross-institutional validation remains limited, raising questions about model transferability across academic systems with divergent evaluation cultures and publication norms.

## Conclusion

This research systematically investigates the application of knowledge graph-enhanced graph neural networks for identifying innovation potential in scientific talents, addressing fundamental limitations of traditional evaluation methods that rely predominantly on static bibliometric indicators. The study establishes a comprehensive methodological framework encompassing heterogeneous academic knowledge graph construction through multi-source data integration, entity relationship extraction and alignment strategies, meta-path-based attention mechanisms for selective information aggregation, and adaptive fusion of semantic knowledge with structural features. Through extensive experiments on real-world academic datasets containing over 128,000 researchers across multiple disciplines, the proposed model demonstrates substantial performance improvements, achieving 85.21% accuracy and 0.9014 AUC-ROC score, representing significant advances over state-of-the-art baseline approaches.

The primary innovations of this work reside in three interconnected dimensions. First, the construction of domain-specific knowledge graphs that capture rich semantic relationships among researchers, publications, institutions, and research topics, extending beyond conventional co-authorship networks to incorporate mentorship relations, project collaborations, and knowledge flow patterns. Second, the design of a novel heterogeneous graph neural network architecture that leverages knowledge graph embeddings to enhance node representations, employs meta-path-based attention to distinguish relationship significance, and implements gated fusion mechanisms to adaptively balance multiple information sources. Third, the establishment of interpretable prediction frameworks through attention weight visualization and gradient-based attribution analysis, addressing critical transparency requirements for practical deployment in talent management systems.

The theoretical contributions of this research advance the fields of computational social science and machine learning through demonstrating effective integration of structured knowledge with data-driven learning paradigms. By bridging knowledge graph representation learning and graph neural network architectures, this work provides generalizable methodologies applicable to diverse domains involving heterogeneous relational data and prospective outcome prediction under uncertainty. The practical significance manifests in enabling more equitable and effective talent identification processes that recognize potential beyond accumulated achievements, particularly benefiting early-career researchers and addressing cold-start problems inherent in conventional evaluation systems. Real-world deployment scenarios encompass university recruitment optimization, research funding allocation, mentorship matching, and strategic workforce planning for research institutions and technology organizations.

Despite these contributions, the current study exhibits limitations that constrain generalization and practical applicability. The dependency on publication-centric data sources may inadequately capture alternative impact pathways including industry collaborations, policy influence, and public engagement activities increasingly valued in contemporary research assessment frameworks. The knowledge graph construction process remains labor-intensive and domain-specific, limiting scalability across diverse disciplinary contexts without substantial adaptation efforts. Furthermore, the model’s interpretability, while enhanced relative to black-box alternatives, requires further development to meet transparency standards for high-stakes decision contexts involving career advancement and resource allocation.

Future research directions should explore several promising avenues to address existing limitations and extend the framework’s capabilities. Incorporating multimodal data sources including textual content from publications, code repositories, and online scholarly discourse could enrich semantic representations and capture nuanced aspects of research contributions. Recent advances in graph-based learning offer promising directions for improving talent assessment models. For instance, multiview fusion strategies that jointly exploit diverse biological or relational network features through fuzzy clustering have shown notable success in complex identification tasks^49^, and these multiview fusion principles could be adapted to integrate heterogeneous academic signals—such as citation networks, collaboration graphs, and topic co-occurrence structures—within a unified clustering-aware framework. Similarly, generative Bayesian approaches to attributed graph clustering^50^ provide principled mechanisms for inferring latent community structure from both node attributes and link topology, which could enhance the identification of research communities and talent clusters in academic networks. Integrating such techniques into our pipeline represents a natural extension that may strengthen both the representational richness and the interpretive depth of talent potential predictions.

Developing continual learning mechanisms to update knowledge graphs and model parameters with streaming data would enable real-time adaptation to evolving research landscapes and emerging collaboration patterns. Investigating fairness-aware learning objectives and bias mitigation strategies remains crucial for ensuring equitable treatment across demographic groups, disciplinary backgrounds, and institutional affiliations. Finally, extending the framework to support multi-objective optimization scenarios that balance innovation potential with diversity, interdisciplinarity, and societal impact considerations would enhance alignment with contemporary science policy priorities and organizational values in the knowledge economy era.

## Supplementary Information

Below is the link to the electronic supplementary material.

Supplementary Material 1

## Data Availability

Two upstream sources underpin the corpus, and each carries its own licensing posture, which we lay out here in some detail to support transparent reuse. Microsoft Academic Graph (MAG) records were retrieved on 15 October 2021 under the Open Data Commons Attribution Licence (ODC-BY 1.0); Microsoft retired the service on 31 December 2021, and the OpenAlex project (https://openalex.org) has since served as a public successor that inherits MAG’s identifier space. Web of Science Core Collection records were obtained through the institutional subscription held by Anyang Vocational and Technical College; Clarivate Analytics’ subscription terms preclude direct redistribution of the underlying records, and we therefore document the access pathway rather than rehosting the raw files.To honour those constraints while still meeting the journal’s reproducibility expectations, an anonymised 500-researcher sample dataset was assembled. The sample preserves the full schema of the production corpus, the relative class balance across high, medium and low innovation-potential categories, and the temporal split that separates the 2010–2018 observation window from the 2019–2021 labelling window. The archive bundles all relational tables (researchers, publications, institutions, topics, authorship, citation, collaboration, affiliation, mentorship, funding and topic-engagement edges), the entity–relation triples used for embedding pre-training, a data dictionary and the complete schema description, together with step-by-step instructions for reconstructing the full corpus from MAG/OpenAlex plus a Web of Science subscription. The processed sample dataset, the supporting documentation and the reproducibility scripts have been packaged and uploaded to Related files. The sample dataset is released under the Creative Commons Attribution 4.0 International Licence (CC-BY-4.0). Any further materials are available from the corresponding author on reasonable request.

## Abbreviations

GNN: Graph Neural Network
GAT: Graph Attention Network
GCN: Graph Convolutional Network
HAN: Heterogeneous Attention Network
MAGNN: Metapath Aggregated Graph Neural Network
KG: Knowledge Graph
HGNN: Heterogeneous Graph Neural Network
KG-HGNN: Knowledge Graph-Enhanced Heterogeneous Graph Neural Network
API: Application Programming Interface
AUC-ROC: Area Under the Receiver Operating Characteristic Curve
NDCG: Normalized Discounted Cumulative Gain
SVM: Support Vector Machine
BERT: Bidirectional Encoder Representations from Transformers
SHAP: SHapley Additive exPlanations

## Code availability

A complete reference implementation of KG-HGNN has been packaged and uploaded to **Related files**. The archive contains the meta-path attention layers, the gated fusion module, the multi-task training loop, the knowledge-graph embedding wrappers (TransE, ComplEx and RotatE), the data-loader utilities and the full evaluation suite that produced the metrics in Tables 7, 8, 9 and 10. Pinned dependency versions—PyTorch 2.0.1, PyTorch Geometric 2.4.0, scikit-learn 1.3.2, DGL-KE 0.1.2, Transformers 4.35.2 and several smaller packages—are listed in *requirements.txt*. Configuration files reproducing every reported run, including the embedding-method comparison in Table 2, the unfreezing schedule in Table 3, the hyperparameter sweep in Table 4 and the loss-coefficient grid in Table 5, are organised under *configs/*, and shell wrappers for the principal experiments are provided under *scripts/*. The source code is released under the MIT licence; the bundled sample dataset under CC-BY-4.0. No access restrictions apply to the code itself, and we welcome questions or pull requests directed to the corresponding author.
