# Supplementary material for: Knowledge graph-enhanced heterogeneous graph neural network for scientific talent innovation potential identification
Source: Sci Rep. 2026 Jun 1;16:25036. doi: 10.1038/s41598-026-54613-x (PMC13462963; doi:10.1038/s41598-026-54613-x)
Supplement: Supplementary file 1 [file 41598_2026_54613_MOESM1_ESM.docx]

**Supplementary File 1: Knowledge Graph-Enhanced Heterogeneous Graph Neural Network for Scientific Talent Innovation Potential Identification**

**Supplementary Tables**

**Table S1. Node Feature Dimensions and Descriptions**

| **Feature Category** | **Feature Name** | **Dimension** | **Description** |
| --- | --- | --- | --- |
| Publication Metrics | Publication count | 1 | Total number of indexed publications |
| Publication Metrics | Citation count | 1 | Total citations received |
| Publication Metrics | H-index | 1 | Hirsch index value |
| Publication Metrics | Average citations per paper | 1 | Mean citation count per publication |
| Publication Metrics | First-author ratio | 1 | Proportion of first-authored papers |
| Publication Metrics | Top venue ratio | 1 | Proportion in top-tier venues |
| Collaboration Metrics | Co-author count | 1 | Number of unique collaborators |
| Collaboration Metrics | Collaboration diversity | 1 | Institutional diversity of collaborators |
| Collaboration Metrics | Betweenness centrality | 1 | Network betweenness measure |
| Collaboration Metrics | Clustering coefficient | 1 | Local clustering coefficient |
| Collaboration Metrics | Structural holes | 1 | Burt's constraint measure |
| Topic Features | Topic entropy | 1 | Research topic diversity |
| Topic Features | Topic evolution velocity | 1 | Rate of topic change over time |
| Topic Features | Interdisciplinary index | 1 | Cross-domain publication ratio |
| Topic Features | Topic embedding | 128 | Averaged topic vector representation |
| Temporal Features | Publication year embedding | 32 | Sinusoidal position encoding |
| Temporal Features | Career trajectory slope | 1 | Linear regression coefficient of output |
| Temporal Features | Citation age decay | 1 | Time-weighted citation impact |
| Temporal Features | Activity recency | 1 | Days since last publication |
| Knowledge Graph Embedding | TransE embedding | 128 | Pre-trained entity embedding |
| **Total** |  | **304** |  |

**Table S2. Meta-path Statistics and Attention Weight Distribution**

| **Meta-path** | **Notation** | **Semantic Meaning** | **Avg. Path Count per Node** | **Learned Attention Weight (β)** |
| --- | --- | --- | --- | --- |
| Researcher-Publication-Researcher | R-P-R | Direct collaboration | 45.7 ± 32.4 | 0.341 ± 0.045 |
| Researcher-Institution-Researcher | R-I-R | Institutional connection | 128.3 ± 89.6 | 0.227 ± 0.038 |
| Researcher-Publication-Citation-Publication-Researcher | R-P-C-P-R | Knowledge influence | 312.5 ± 187.2 | 0.189 ± 0.031 |
| Researcher-Topic-Researcher | R-T-R | Research interest similarity | 89.4 ± 56.8 | 0.112 ± 0.024 |
| Researcher-Funding-Researcher | R-F-R | Project collaboration | 12.3 ± 8.9 | 0.078 ± 0.019 |
| Researcher-Publication-Topic-Publication-Researcher | R-P-T-P-R | Cross-domain transfer | 156.7 ± 112.4 | 0.053 ± 0.015 |

**Table S3. Knowledge Graph Triple Examples (Anonymized)**

| **Head Entity** | **Relation** | **Tail Entity** | **Temporal Attribute** |
| --- | --- | --- | --- |
| Researcher_CS_0001 | authored | Publication_2015_0234 | 2015-03-15 |
| Researcher_CS_0001 | collaborates_with | Researcher_CS_0089 | 2014-2018 |
| Publication_2015_0234 | cites | Publication_2013_1456 | 2015-03-15 |
| Researcher_CS_0001 | affiliated_with | Institution_0012 | 2012-2019 |
| Researcher_CS_0001 | mentored_by | Researcher_CS_0003 | 2010-2014 |
| Researcher_CS_0001 | participates_in | Funding_NSF_0078 | 2016-2019 |
| Researcher_CS_0001 | researches | Topic_MachineLearning | 2012-2018 |
| Publication_2015_0234 | published_in | Venue_NeurIPS | 2015 |
| Institution_0012 | located_in | Country_USA | - |
| Topic_MachineLearning | related_to | Topic_DeepLearning | - |

**Table S4. Dataset Split Statistics**

| **Split** | **Researchers** | **Publications** | **High-Potential** | **Medium-Potential** | **Low-Potential** |
| --- | --- | --- | --- | --- | --- |
| Training (70%) | 89,919 | 872,048 | 12,966 (14.4%) | 50,019 (55.6%) | 26,934 (30.0%) |
| Validation (15%) | 19,268 | 186,867 | 2,778 (14.4%) | 10,718 (55.6%) | 5,772 (30.0%) |
| Test (15%) | 19,269 | 186,868 | 2,779 (14.4%) | 10,719 (55.6%) | 5,771 (30.0%) |
| **Total** | **128,456** | **1,245,783** | **18,523** | **71,456** | **38,477** |

**Table S5. Innovation Potential Labeling Criteria**

| **Label** | **Criteria** | **Threshold** | **Researchers Count** |
| --- | --- | --- | --- |
| High-Potential | Composite score in top 15% | Score ≥ 0.78 | 18,523 |
| Medium-Potential | Composite score in middle 60% | 0.35 ≤ Score < 0.78 | 71,456 |
| Low-Potential | Composite score in bottom 25% | Score < 0.35 | 38,477 |

**Composite Score Formula:** $$S_{composite} = 0.30 \times \Delta h_{norm} + 0.25 \times G_{cite,norm} + 0.20 \times E_{collab,norm} + 0.15 \times B_{pub,norm} + 0.10 \times D_{topic,norm}$$

Where:

- $\Delta h_{norm}$: Normalized h-index increment (2019-2021)
- $G_{cite,norm}$: Normalized citation growth rate
- $E_{collab,norm}$: Normalized collaboration network expansion
- $B_{pub,norm}$: Normalized breakthrough publication frequency
- $D_{topic,norm}$: Normalized topic diversity change

**Table S6. Hyperparameter Search Space and Final Configuration**

| **Component** | **Parameter** | **Search Space** | **Final Value** | **Selection Method** |
| --- | --- | --- | --- | --- |
| KG Embedding | Embedding dimension | {64, 128, 256} | 128 | Grid search |
| KG Embedding | TransE margin | {0.5, 1.0, 2.0} | 1.0 | Grid search |
| KG Embedding | Negative samples | {5, 10, 20} | 10 | Grid search |
| GNN Architecture | Hidden dimension | {64, 128, 256, 512} | 256 | Grid search |
| GNN Architecture | Number of layers | {2, 3, 4, 5} | 3 | Grid search |
| GNN Architecture | Attention heads | {4, 8, 16} | 8 | Grid search |
| GNN Architecture | Aggregation function | {mean, sum, max} | mean | Grid search |
| Optimization | Learning rate | {1e-4, 5e-4, 1e-3, 5e-3} | 1e-3 | Grid search |
| Optimization | Batch size | {32, 64, 128, 256} | 128 | Grid search |
| Optimization | Weight decay | {1e-5, 1e-4, 1e-3} | 1e-4 | Grid search |
| Optimization | Optimizer | {Adam, AdamW, SGD} | Adam | Grid search |
| Regularization | Dropout rate | {0.1, 0.2, 0.3, 0.4, 0.5} | 0.3 | Grid search |
| Regularization | Label smoothing | {0.0, 0.1, 0.2} | 0.1 | Grid search |
| Loss Weights | λ₁ (reconstruction) | {0.05, 0.1, 0.2} | 0.1 | Grid search |
| Loss Weights | λ₂ (link prediction) | {0.01, 0.05, 0.1} | 0.05 | Grid search |
| Training | Max epochs | {100, 200, 300} | 200 | Early stopping |
| Training | Early stopping patience | {10, 20, 30} | 20 | Fixed |
| Training | KG unfreeze epoch | {30, 50, 70} | 50 | Grid search |
| Training | KG fine-tune LR ratio | {0.01, 0.1, 0.5} | 0.1 | Grid search |

**Table S7. Discipline-Specific Performance Breakdown**

| **Discipline** | **Researchers** | **Accuracy** | **Precision** | **Recall** | **F1-Score** | **AUC-ROC** |
| --- | --- | --- | --- | --- | --- | --- |
| Computer Science | 42,156 | 0.8672 ± 0.008 | 0.8534 ± 0.010 | 0.8421 ± 0.012 | 0.8477 ± 0.009 | 0.9156 ± 0.006 |
| Engineering | 38,234 | 0.8456 ± 0.009 | 0.8312 ± 0.011 | 0.8198 ± 0.013 | 0.8255 ± 0.010 | 0.8967 ± 0.007 |
| Life Sciences | 31,456 | 0.8312 ± 0.010 | 0.8178 ± 0.012 | 0.8034 ± 0.014 | 0.8105 ± 0.011 | 0.8823 ± 0.008 |
| Physics | 16,610 | 0.8234 ± 0.012 | 0.8089 ± 0.014 | 0.7956 ± 0.016 | 0.8022 ± 0.013 | 0.8745 ± 0.009 |
| **Overall** | **128,456** | **0.8521 ± 0.007** | **0.8392 ± 0.009** | **0.8267 ± 0.011** | **0.8329 ± 0.008** | **0.9014 ± 0.006** |

**Table S8. Career Stage-Specific Performance Breakdown**

| **Career Stage** | **Years Since First Pub.** | **Researchers** | **Accuracy** | **F1-Score** | **AUC-ROC** |
| --- | --- | --- | --- | --- | --- |
| Early-Career | 0-5 years | 41,234 | 0.8234 ± 0.011 | 0.8067 ± 0.013 | 0.8756 ± 0.009 |
| Mid-Career | 6-12 years | 52,891 | 0.8623 ± 0.008 | 0.8478 ± 0.010 | 0.9134 ± 0.006 |
| Senior | >12 years | 34,331 | 0.8712 ± 0.009 | 0.8567 ± 0.011 | 0.9189 ± 0.007 |

**Table S9. Entity Disambiguation Performance**

| **Entity Type** | **Method** | **Precision** | **Recall** | **F1-Score** | **Test Set Size** |
| --- | --- | --- | --- | --- | --- |
| Researcher | Multi-feature matching + SciBERT | 0.943 | 0.917 | 0.930 | 5,000 pairs |
| Institution | Hierarchical clustering + name normalization | 0.962 | 0.951 | 0.956 | 2,000 records |
| Publication | DOI + metadata matching | 0.987 | 0.992 | 0.989 | 10,000 records |
| Research Topic | Keyword co-occurrence + LDA | 0.891 | 0.876 | 0.883 | 3,000 topics |

**Table S10. Mentorship Relation Extraction Performance**

| **Source** | **Extraction Method** | **Precision** | **Recall** | **Sample Size** |
| --- | --- | --- | --- | --- |
| Mathematics Genealogy Project | Direct database query | 0.985 | 0.923 | 45,678 |
| AI Genealogy | Direct database query | 0.978 | 0.912 | 23,456 |
| Dissertation acknowledgments | Pattern matching rules | 0.876 | 0.734 | 118,298 |
| **Combined** | Ensemble with conflict resolution | 0.921 | 0.847 | 187,432 |

**Table S11. Feature Importance Ranking (SHAP Values)**

| Rank | Feature | Mean |SHAP| | Std |SHAP| | Feature Category | |------|---------|-------------|------------|------------------| | 1 | Collaboration diversity | 0.0823 | 0.0156 | Collaboration | | 2 | Topic entropy | 0.0756 | 0.0143 | Topic | | 3 | Betweenness centrality | 0.0689 | 0.0132 | Collaboration | | 4 | H-index growth rate | 0.0634 | 0.0121 | Publication | | 5 | Structural holes | 0.0578 | 0.0118 | Collaboration | | 6 | Top venue ratio | 0.0523 | 0.0109 | Publication | | 7 | Topic evolution velocity | 0.0489 | 0.0098 | Topic | | 8 | Interdisciplinary index | 0.0456 | 0.0092 | Topic | | 9 | Citation growth rate | 0.0423 | 0.0087 | Publication | | 10 | First-author ratio | 0.0389 | 0.0081 | Publication |

**Table S12. Cross-Validation Results (10-Fold)**

| **Fold** | **Accuracy** | **Precision** | **Recall** | **F1-Score** | **AUC-ROC** |
| --- | --- | --- | --- | --- | --- |
| 1 | 0.8534 | 0.8401 | 0.8278 | 0.8339 | 0.9023 |
| 2 | 0.8512 | 0.8378 | 0.8256 | 0.8317 | 0.9008 |
| 3 | 0.8498 | 0.8365 | 0.8241 | 0.8303 | 0.8998 |
| 4 | 0.8567 | 0.8432 | 0.8312 | 0.8372 | 0.9056 |
| 5 | 0.8489 | 0.8356 | 0.8234 | 0.8295 | 0.8989 |
| 6 | 0.8523 | 0.8389 | 0.8267 | 0.8328 | 0.9012 |
| 7 | 0.8545 | 0.8412 | 0.8289 | 0.8350 | 0.9034 |
| 8 | 0.8501 | 0.8367 | 0.8245 | 0.8306 | 0.8996 |
| 9 | 0.8478 | 0.8345 | 0.8223 | 0.8284 | 0.8978 |
| 10 | 0.8563 | 0.8428 | 0.8306 | 0.8367 | 0.9048 |
| **Mean** | **0.8521** | **0.8387** | **0.8265** | **0.8326** | **0.9014** |
| **Std** | **0.0029** | **0.0031** | **0.0030** | **0.0030** | **0.0025** |

**Table S13. Statistical Significance Tests (Paired t-test vs. Baselines)**

| **Baseline Model** | **Δ Accuracy** | **t-statistic** | **p-value** | **Effect Size (Cohen's d)** |
| --- | --- | --- | --- | --- |
| Random Forest | +0.1697 | 42.56 | <0.0001 | 4.85 |
| Gradient Boosting | +0.1365 | 38.23 | <0.0001 | 4.12 |
| SVM | +0.1576 | 40.89 | <0.0001 | 4.56 |
| GCN | +0.1132 | 31.45 | <0.0001 | 3.24 |
| GAT | +0.0997 | 28.67 | <0.0001 | 2.89 |
| HAN | +0.0629 | 19.34 | <0.0001 | 1.98 |
| MAGNN | +0.0508 | 15.78 | <0.0001 | 1.63 |

**Table S14. Model Calibration Results**

| **Confidence Bin** | **Predicted Probability** | **Actual Accuracy** | **Sample Count** | **ECE Contribution** |
| --- | --- | --- | --- | --- |
| 0.0-0.1 | 0.052 | 0.048 | 4,234 | 0.0003 |
| 0.1-0.2 | 0.156 | 0.149 | 5,678 | 0.0004 |
| 0.2-0.3 | 0.251 | 0.243 | 6,123 | 0.0005 |
| 0.3-0.4 | 0.348 | 0.339 | 7,456 | 0.0005 |
| 0.4-0.5 | 0.452 | 0.461 | 8,234 | 0.0007 |
| 0.5-0.6 | 0.553 | 0.548 | 9,123 | 0.0005 |
| 0.6-0.7 | 0.651 | 0.643 | 10,456 | 0.0008 |
| 0.7-0.8 | 0.748 | 0.756 | 12,345 | 0.0010 |
| 0.8-0.9 | 0.852 | 0.847 | 15,678 | 0.0008 |
| 0.9-1.0 | 0.945 | 0.951 | 48,129 | 0.0029 |
| **Total ECE** |  |  | **128,456** | **0.0084** |

**Table S15. Real-World Deployment Validation Results**

| **University** | **Department** | **Candidates** | **Model Top-10** | **Expert Top-10** | **NDCG Overlap** | **Agreement Rate** |
| --- | --- | --- | --- | --- | --- | --- |
| University X | Computer Science | 456 | 10 | 10 | 0.823 | 80.0% |
| University X | Engineering | 389 | 10 | 10 | 0.789 | 77.0% |
| University Y | Life Sciences | 534 | 10 | 10 | 0.756 | 75.0% |
| University Y | Physics | 287 | 10 | 10 | 0.778 | 76.0% |
| University Z | Computer Science | 412 | 10 | 10 | 0.834 | 81.0% |
| University Z | Engineering | 378 | 10 | 10 | 0.801 | 79.0% |
| University Z | Life Sciences | 391 | 10 | 10 | 0.767 | 76.0% |
| **Total** |  | **2,847** |  |  | **0.784** | **78.4%** |

**Supplementary Code**

**Code S1. KG-HGNN Model Implementation**

import torch

import torch.nn as nn

import torch.nn.functional as F

from torch_geometric.nn import MessagePassing

from torch_geometric.utils import softmax

import numpy as np

class MetaPathAttention(nn.Module):

"""Meta-path based attention mechanism for heterogeneous graphs."""

def __init__(self, in_dim, out_dim, num_heads=8, dropout=0.3):

super(MetaPathAttention, self).__init__()

self.num_heads = num_heads

self.out_dim = out_dim

self.head_dim = out_dim // num_heads

self.W = nn.Linear(in_dim, out_dim, bias=False)

self.a = nn.Parameter(torch.zeros(1, num_heads, 2 * self.head_dim))

nn.init.xavier_uniform_(self.a)

self.dropout = nn.Dropout(dropout)

self.leaky_relu = nn.LeakyReLU(0.2)

def forward(self, h, edge_index):

"""

Args:

h: Node features [N, in_dim]

edge_index: Edge indices [2, E]

Returns:

Updated node features [N, out_dim]

"""

N = h.size(0)

h_transformed = self.W(h).view(N, self.num_heads, self.head_dim)

src, dst = edge_index

h_src = h_transformed[src] # [E, num_heads, head_dim]

h_dst = h_transformed[dst] # [E, num_heads, head_dim]

\# Compute attention coefficients

edge_features = torch.cat([h_src, h_dst], dim=-1) # [E, num_heads, 2*head_dim]

attn_logits = (edge_features * self.a).sum(dim=-1) # [E, num_heads]

attn_logits = self.leaky_relu(attn_logits)

\# Softmax over neighbors

attn_weights = softmax(attn_logits, dst, num_nodes=N) # [E, num_heads]

attn_weights = self.dropout(attn_weights)

\# Aggregate

out = torch.zeros(N, self.num_heads, self.head_dim, device=h.device)

attn_weights = attn_weights.unsqueeze(-1) # [E, num_heads, 1]

weighted_src = h_src * attn_weights # [E, num_heads, head_dim]

out.scatter_add_(0, dst.unsqueeze(-1).unsqueeze(-1).expand_as(weighted_src), weighted_src)

return out.view(N, self.out_dim)

class SemanticAttention(nn.Module):

"""Semantic-level attention for aggregating meta-path representations."""

def __init__(self, hidden_dim):

super(SemanticAttention, self).__init__()

self.W_s = nn.Linear(hidden_dim, hidden_dim)

self.q = nn.Parameter(torch.zeros(hidden_dim))

nn.init.xavier_uniform_(self.q.unsqueeze(0))

def forward(self, z_list):

"""

Args:

z_list: List of meta-path representations, each [N, hidden_dim]

Returns:

Fused representation [N, hidden_dim]

Attention weights [num_meta_paths]

"""

\# Stack meta-path representations: [num_meta_paths, N, hidden_dim]

z_stack = torch.stack(z_list, dim=0)

\# Compute semantic attention scores

w = torch.tanh(self.W_s(z_stack)) # [num_meta_paths, N, hidden_dim]

scores = (w * self.q).sum(dim=-1).mean(dim=1) # [num_meta_paths]

beta = F.softmax(scores, dim=0) # [num_meta_paths]

\# Weighted aggregation

beta = beta.view(-1, 1, 1) # [num_meta_paths, 1, 1]

z_fused = (beta * z_stack).sum(dim=0) # [N, hidden_dim]

return z_fused, beta.squeeze()

class GatedFusion(nn.Module):

"""Gated fusion mechanism for combining structural and KG features."""

def __init__(self, hidden_dim):

super(GatedFusion, self).__init__()

self.W_g = nn.Linear(2 * hidden_dim, hidden_dim)

def forward(self, h_struct, h_kg):

"""

Args:

h_struct: Structural embeddings [N, hidden_dim]

h_kg: Knowledge graph embeddings [N, hidden_dim]

Returns:

Fused embeddings [N, hidden_dim]

"""

combined = torch.cat([h_struct, h_kg], dim=-1)

gate = torch.sigmoid(self.W_g(combined))

h_fused = gate * h_struct + (1 - gate) * h_kg

return h_fused

class KGHGNN(nn.Module):

"""Knowledge Graph-Enhanced Heterogeneous Graph Neural Network."""

def __init__(self, config):

super(KGHGNN, self).__init__()

self.input_dim = config['input_dim']

self.hidden_dim = config['hidden_dim']

self.num_classes = config['num_classes']

self.num_layers = config['num_layers']

self.num_heads = config['num_heads']

self.num_meta_paths = config['num_meta_paths']

self.dropout = config['dropout']

self.kg_dim = config['kg_dim']

\# Input projection

self.input_proj = nn.Linear(self.input_dim + self.kg_dim, self.hidden_dim)

\# Meta-path attention layers for each meta-path type

self.meta_path_layers = nn.ModuleList([

nn.ModuleList([

MetaPathAttention(self.hidden_dim, self.hidden_dim,

self.num_heads, self.dropout)

for _ in range(self.num_layers)

])

for _ in range(self.num_meta_paths)

])

\# Semantic attention

self.semantic_attention = SemanticAttention(self.hidden_dim)

\# Gated fusion

self.gated_fusion = GatedFusion(self.hidden_dim)

\# KG embedding projection

self.kg_proj = nn.Linear(self.kg_dim, self.hidden_dim)

\# Classifier

self.classifier = nn.Sequential(

nn.Linear(self.hidden_dim, self.hidden_dim // 2),

nn.ReLU(),

nn.Dropout(self.dropout),

nn.Linear(self.hidden_dim // 2, self.num_classes)

)

\# Reconstruction decoder (for auxiliary loss)

self.decoder = nn.Linear(self.hidden_dim, self.input_dim)

self.dropout_layer = nn.Dropout(self.dropout)

def forward(self, x, kg_emb, meta_path_edge_indices):

"""

Args:

x: Node features [N, input_dim]

kg_emb: Pre-trained KG embeddings [N, kg_dim]

meta_path_edge_indices: List of edge indices for each meta-path

Returns:

logits: Classification logits [N, num_classes]

h_fused: Fused node representations [N, hidden_dim]

x_recon: Reconstructed features [N, input_dim]

beta: Semantic attention weights [num_meta_paths]

"""

\# Initialize node representations

h = torch.cat([x, kg_emb], dim=-1)

h = self.input_proj(h)

h = F.relu(h)

h = self.dropout_layer(h)

\# Meta-path based aggregation

z_list = []

for mp_idx, edge_index in enumerate(meta_path_edge_indices):

z = h

for layer in self.meta_path_layers[mp_idx]:

z = layer(z, edge_index)

z = F.elu(z)

z = self.dropout_layer(z)

z_list.append(z)

\# Semantic-level attention fusion

h_struct, beta = self.semantic_attention(z_list)

\# Project KG embeddings

h_kg = self.kg_proj(kg_emb)

\# Gated fusion

h_fused = self.gated_fusion(h_struct, h_kg)

\# Classification

logits = self.classifier(h_fused)

\# Reconstruction

x_recon = self.decoder(h_fused)

return logits, h_fused, x_recon, beta

class KGHGNNTrainer:

"""Training and evaluation utilities for KG-HGNN."""

def __init__(self, model, config, device):

self.model = model.to(device)

self.device = device

self.config = config

self.optimizer = torch.optim.Adam(

model.parameters(),

lr=config['learning_rate'],

weight_decay=config['weight_decay']

)

self.scheduler = torch.optim.lr_scheduler.ReduceLROnPlateau(

self.optimizer, mode='max', factor=0.5, patience=10

)

self.criterion = nn.CrossEntropyLoss(label_smoothing=config.get('label_smoothing', 0.1))

def compute_loss(self, logits, labels, x, x_recon, h_fused, edge_index):

"""Compute multi-task loss."""

\# Classification loss

loss_cls = self.criterion(logits, labels)

\# Reconstruction loss

loss_recon = F.mse_loss(x_recon, x)

\# Link prediction loss

src, dst = edge_index

pos_score = (h_fused[src] * h_fused[dst]).sum(dim=-1)

\# Negative sampling

neg_dst = torch.randint(0, h_fused.size(0), (src.size(0),), device=self.device)

neg_score = (h_fused[src] * h_fused[neg_dst]).sum(dim=-1)

loss_link = -torch.log(torch.sigmoid(pos_score) + 1e-8).mean() \

- torch.log(1 - torch.sigmoid(neg_score) + 1e-8).mean()

\# Total loss

lambda1 = self.config.get('lambda1', 0.1)

lambda2 = self.config.get('lambda2', 0.05)

total_loss = loss_cls + lambda1 * loss_recon + lambda2 * loss_link

return total_loss, loss_cls, loss_recon, loss_link

def train_epoch(self, data_loader):

"""Train for one epoch."""

self.model.train()

total_loss = 0

for batch in data_loader:

batch = {k: v.to(self.device) for k, v in batch.items()}

self.optimizer.zero_grad()

logits, h_fused, x_recon, beta = self.model(

batch['x'], batch['kg_emb'], batch['meta_path_edges']

)

loss, _, _, _ = self.compute_loss(

logits, batch['labels'], batch['x'],

x_recon, h_fused, batch['edge_index']

)

loss.backward()

torch.nn.utils.clip_grad_norm_(self.model.parameters(), 1.0)

self.optimizer.step()

total_loss += loss.item()

return total_loss / len(data_loader)

@torch.no_grad()

def evaluate(self, data_loader):

"""Evaluate model performance."""

self.model.eval()

all_preds = []

all_labels = []

all_probs = []

for batch in data_loader:

batch = {k: v.to(self.device) for k, v in batch.items()}

logits, _, _, _ = self.model(

batch['x'], batch['kg_emb'], batch['meta_path_edges']

)

probs = F.softmax(logits, dim=-1)

preds = logits.argmax(dim=-1)

all_preds.append(preds.cpu())

all_labels.append(batch['labels'].cpu())

all_probs.append(probs.cpu())

all_preds = torch.cat(all_preds)

all_labels = torch.cat(all_labels)

all_probs = torch.cat(all_probs)

\# Compute metrics

accuracy = (all_preds == all_labels).float().mean().item()

return {

'accuracy': accuracy,

'predictions': all_preds,

'labels': all_labels,

'probabilities': all_probs

}

**Code S2. Knowledge Graph Embedding (TransE)**

import torch

import torch.nn as nn

import torch.nn.functional as F

from torch.utils.data import DataLoader, Dataset

import numpy as np

class TransE(nn.Module):

"""TransE knowledge graph embedding model."""

def __init__(self, num_entities, num_relations, embedding_dim, margin=1.0):

super(TransE, self).__init__()

self.num_entities = num_entities

self.num_relations = num_relations

self.embedding_dim = embedding_dim

self.margin = margin

\# Entity and relation embeddings

self.entity_embeddings = nn.Embedding(num_entities, embedding_dim)

self.relation_embeddings = nn.Embedding(num_relations, embedding_dim)

\# Initialize embeddings

nn.init.xavier_uniform_(self.entity_embeddings.weight)

nn.init.xavier_uniform_(self.relation_embeddings.weight)

\# Normalize relation embeddings

self.relation_embeddings.weight.data = F.normalize(

self.relation_embeddings.weight.data, p=2, dim=1

)

def forward(self, heads, relations, tails):

"""

Compute TransE scores for given triples.

Args:

heads: Head entity indices [batch_size]

relations: Relation indices [batch_size]

tails: Tail entity indices [batch_size]

Returns:

scores: Distance scores [batch_size]

"""

h = self.entity_embeddings(heads)

r = self.relation_embeddings(relations)

t = self.entity_embeddings(tails)

\# L2 distance: ||h + r - t||

scores = torch.norm(h + r - t, p=2, dim=1)

return scores

def get_entity_embeddings(self, entity_ids=None):

"""Get entity embeddings."""

if entity_ids is None:

return self.entity_embeddings.weight.data

return self.entity_embeddings(entity_ids)

class KGTripleDataset(Dataset):

"""Dataset for knowledge graph triples."""

def __init__(self, triples, num_entities, num_neg_samples=10):

self.triples = triples # [(h, r, t), ...]

self.num_entities = num_entities

self.num_neg_samples = num_neg_samples

def __len__(self):

return len(self.triples)

def __getitem__(self, idx):

h, r, t = self.triples[idx]

\# Generate negative samples by corrupting head or tail

neg_tails = np.random.randint(0, self.num_entities, self.num_neg_samples)

return {

'head': torch.tensor(h, dtype=torch.long),

'relation': torch.tensor(r, dtype=torch.long),

'tail': torch.tensor(t, dtype=torch.long),

'neg_tails': torch.tensor(neg_tails, dtype=torch.long)

}

class TransETrainer:

"""Trainer for TransE model."""

def __init__(self, model, config, device):

self.model = model.to(device)

self.device = device

self.config = config

self.optimizer = torch.optim.Adam(

model.parameters(),

lr=config['learning_rate']

)

def margin_loss(self, pos_scores, neg_scores):

"""Compute margin-based ranking loss."""

return torch.relu(self.model.margin + pos_scores - neg_scores).mean()

def train_epoch(self, data_loader):

"""Train for one epoch."""

self.model.train()

total_loss = 0

for batch in data_loader:

batch = {k: v.to(self.device) for k, v in batch.items()}

self.optimizer.zero_grad()

\# Positive scores

pos_scores = self.model(

batch['head'], batch['relation'], batch['tail']

)

\# Negative scores

batch_size = batch['head'].size(0)

num_neg = batch['neg_tails'].size(1)

heads_expanded = batch['head'].unsqueeze(1).expand(-1, num_neg).reshape(-1)

relations_expanded = batch['relation'].unsqueeze(1).expand(-1, num_neg).reshape(-1)

neg_tails_flat = batch['neg_tails'].reshape(-1)

neg_scores = self.model(

heads_expanded, relations_expanded, neg_tails_flat

).view(batch_size, num_neg).mean(dim=1)

loss = self.margin_loss(pos_scores, neg_scores)

loss.backward()

\# Normalize entity embeddings

self.model.entity_embeddings.weight.data = F.normalize(

self.model.entity_embeddings.weight.data, p=2, dim=1

)

self.optimizer.step()

total_loss += loss.item()

return total_loss / len(data_loader)

**Code S3. Entity Disambiguation Module**

import torch

import numpy as np

from transformers import AutoTokenizer, AutoModel

from sklearn.metrics.pairwise import cosine_similarity

import jellyfish

class EntityDisambiguator:

"""Multi-feature entity disambiguation for researchers."""

def __init__(self, scibert_model='allenai/scibert_scivocab_uncased'):

self.tokenizer = AutoTokenizer.from_pretrained(scibert_model)

self.model = AutoModel.from_pretrained(scibert_model)

self.model.eval()

\# Feature weights for disambiguation

self.weights = {

'name_similarity': 0.25,

'affiliation_overlap': 0.20,

'coauthor_overlap': 0.20,

'topic_similarity': 0.20,

'embedding_similarity': 0.15

}

def compute_name_similarity(self, name1, name2):

"""Compute Jaro-Winkler similarity between names."""

return jellyfish.jaro_winkler_similarity(

name1.lower().strip(),

name2.lower().strip()

)

def compute_affiliation_overlap(self, affiliations1, affiliations2):

"""Compute Jaccard overlap of affiliation histories."""

set1 = set(affiliations1)

set2 = set(affiliations2)

if not set1 or not set2:

return 0.0

intersection = len(set1 & set2)

union = len(set1 | set2)

return intersection / union if union > 0 else 0.0

def compute_coauthor_overlap(self, coauthors1, coauthors2):

"""Compute Jaccard overlap of co-author networks."""

set1 = set(coauthors1)

set2 = set(coauthors2)

if not set1 or not set2:

return 0.0

intersection = len(set1 & set2)

union = len(set1 | set2)

return intersection / union if union > 0 else 0.0

def compute_topic_similarity(self, topics1, topics2):

"""Compute cosine similarity of topic distributions."""

if not topics1 or not topics2:

return 0.0

\# Create topic vectors

all_topics = list(set(topics1.keys()) | set(topics2.keys()))

vec1 = np.array([topics1.get(t, 0) for t in all_topics])

vec2 = np.array([topics2.get(t, 0) for t in all_topics])

norm1 = np.linalg.norm(vec1)

norm2 = np.linalg.norm(vec2)

if norm1 == 0 or norm2 == 0:

return 0.0

return np.dot(vec1, vec2) / (norm1 * norm2)

@torch.no_grad()

def get_scibert_embedding(self, text):

"""Get SciBERT embedding for text."""

inputs = self.tokenizer(

text, return_tensors='pt',

max_length=512, truncation=True, padding=True

)

outputs = self.model(**inputs)

\# Use [CLS] token embedding

return outputs.last_hidden_state[:, 0, :].numpy()

def compute_embedding_similarity(self, profile1, profile2):

"""Compute embedding similarity of research profiles."""

\# Concatenate name, affiliations, and sample publications

text1 = f"{profile1['name']} {' '.join(profile1.get('affiliations', []))} " \

f"{' '.join(profile1.get('paper_titles', [])[:5])}"

text2 = f"{profile2['name']} {' '.join(profile2.get('affiliations', []))} " \

f"{' '.join(profile2.get('paper_titles', [])[:5])}"

emb1 = self.get_scibert_embedding(text1)

emb2 = self.get_scibert_embedding(text2)

return cosine_similarity(emb1, emb2)[0, 0]

def compute_disambiguation_score(self, profile1, profile2):

"""

Compute overall disambiguation score between two researcher profiles.

Returns:

score: Float in [0, 1], higher means more likely same person

"""

scores = {

'name_similarity': self.compute_name_similarity(

profile1['name'], profile2['name']

),

'affiliation_overlap': self.compute_affiliation_overlap(

profile1.get('affiliations', []),

profile2.get('affiliations', [])

),

'coauthor_overlap': self.compute_coauthor_overlap(

profile1.get('coauthors', []),

profile2.get('coauthors', [])

),

'topic_similarity': self.compute_topic_similarity(

profile1.get('topics', {}),

profile2.get('topics', {})

),

'embedding_similarity': self.compute_embedding_similarity(

profile1, profile2

)

}

\# Weighted combination

final_score = sum(

self.weights[k] * scores[k] for k in scores

)

return final_score, scores

def is_same_entity(self, profile1, profile2, threshold=0.75):

"""Determine if two profiles refer to the same researcher."""

score, _ = self.compute_disambiguation_score(profile1, profile2)

return score >= threshold

**Code S4. Evaluation Metrics**

import numpy as np

from sklearn.metrics import (

accuracy_score, precision_score, recall_score, f1_score,

roc_auc_score, confusion_matrix, classification_report

)

from scipy import stats

def compute_classification_metrics(y_true, y_pred, y_prob=None, num_classes=3):

"""

Compute comprehensive classification metrics.

Args:

y_true: Ground truth labels

y_pred: Predicted labels

y_prob: Predicted probabilities (optional)

num_classes: Number of classes

Returns:

Dictionary of metrics

"""

metrics = {

'accuracy': accuracy_score(y_true, y_pred),

'precision_macro': precision_score(y_true, y_pred, average='macro'),

'recall_macro': recall_score(y_true, y_pred, average='macro'),

'f1_macro': f1_score(y_true, y_pred, average='macro'),

'precision_weighted': precision_score(y_true, y_pred, average='weighted'),

'recall_weighted': recall_score(y_true, y_pred, average='weighted'),

'f1_weighted': f1_score(y_true, y_pred, average='weighted'),

}

\# Per-class metrics

for cls in range(num_classes):

y_true_binary = (y_true == cls).astype(int)

y_pred_binary = (y_pred == cls).astype(int)

metrics[f'precision_class_{cls}'] = precision_score(y_true_binary, y_pred_binary)

metrics[f'recall_class_{cls}'] = recall_score(y_true_binary, y_pred_binary)

metrics[f'f1_class_{cls}'] = f1_score(y_true_binary, y_pred_binary)

\# AUC-ROC if probabilities provided

if y_prob is not None:

try:

if num_classes == 2:

metrics['auc_roc'] = roc_auc_score(y_true, y_prob[:, 1])

else:

metrics['auc_roc'] = roc_auc_score(

y_true, y_prob, multi_class='ovr', average='weighted'

)

except Exception:

metrics['auc_roc'] = None

\# Confusion matrix

metrics['confusion_matrix'] = confusion_matrix(y_true, y_pred)

return metrics

def compute_ndcg(y_true, y_scores, k=10):

"""

Compute Normalized Discounted Cumulative Gain at k.

Args:

y_true: Ground truth relevance scores

y_scores: Predicted scores

k: Number of top items to consider

Returns:

NDCG@k score

"""

\# Sort by predicted scores

order = np.argsort(y_scores)[::-1][:k]

gains = y_true[order]

\# DCG

discounts = np.log2(np.arange(len(gains)) + 2)

dcg = np.sum(gains / discounts)

\# IDCG (ideal DCG)

ideal_gains = np.sort(y_true)[::-1][:k]

idcg = np.sum(ideal_gains / discounts[:len(ideal_gains)])

if idcg == 0:

return 0.0

return dcg / idcg

def compute_expected_calibration_error(y_true, y_prob, n_bins=10):

"""

Compute Expected Calibration Error.

Args:

y_true: Ground truth labels (binary or multi-class)

y_prob: Predicted probabilities

n_bins: Number of bins for calibration

Returns:

ECE score and per-bin statistics

"""

if len(y_prob.shape) > 1:

\# Multi-class: use max probability

confidences = np.max(y_prob, axis=1)

predictions = np.argmax(y_prob, axis=1)

accuracies = (predictions == y_true).astype(float)

else:

confidences = y_prob

predictions = (y_prob >= 0.5).astype(int)

accuracies = (predictions == y_true).astype(float)

bin_boundaries = np.linspace(0, 1, n_bins + 1)

ece = 0.0

bin_stats = []

for i in range(n_bins):

in_bin = (confidences > bin_boundaries[i]) & (confidences <= bin_boundaries[i + 1])

prop_in_bin = in_bin.mean()

if prop_in_bin > 0:

avg_confidence = confidences[in_bin].mean()

avg_accuracy = accuracies[in_bin].mean()

bin_ece = np.abs(avg_accuracy - avg_confidence) * prop_in_bin

ece += bin_ece

bin_stats.append({

'bin_lower': bin_boundaries[i],

'bin_upper': bin_boundaries[i + 1],

'avg_confidence': avg_confidence,

'avg_accuracy': avg_accuracy,

'count': in_bin.sum(),

'ece_contribution': bin_ece

})

return ece, bin_stats

def paired_ttest(scores1, scores2, alpha=0.05):

"""

Perform paired t-test between two sets of scores.

Args:

scores1: Scores from model 1

scores2: Scores from model 2

alpha: Significance level

Returns:

Test results dictionary

"""

t_stat, p_value = stats.ttest_rel(scores1, scores2)

\# Effect size (Cohen's d)

diff = scores1 - scores2

cohens_d = np.mean(diff) / np.std(diff, ddof=1)

return {

't_statistic': t_stat,

'p_value': p_value,

'significant': p_value < alpha,

'cohens_d': cohens_d,

'mean_diff': np.mean(diff),

'std_diff': np.std(diff, ddof=1)

}

def bootstrap_confidence_interval(metric_func, y_true, y_pred, n_bootstrap=1000, ci=0.95):

"""

Compute bootstrap confidence interval for a metric.

Args:

metric_func: Function to compute metric

y_true: Ground truth

y_pred: Predictions

n_bootstrap: Number of bootstrap samples

ci: Confidence interval level

Returns:

Point estimate and confidence interval

"""

n = len(y_true)

scores = []

for _ in range(n_bootstrap):

indices = np.random.choice(n, n, replace=True)

score = metric_func(y_true[indices], y_pred[indices])

scores.append(score)

scores = np.array(scores)

alpha = (1 - ci) / 2

lower = np.percentile(scores, alpha * 100)

upper = np.percentile(scores, (1 - alpha) * 100)

return {

'mean': np.mean(scores),

'std': np.std(scores),

'ci_lower': lower,

'ci_upper': upper

}

**Code S5. Configuration File**

\# config.py - Model and training configuration

CONFIG = {

\# Data configuration

'data': {

'observation_start': '2010-01-01',

'observation_end': '2018-12-31',

'label_start': '2019-01-01',

'label_end': '2021-12-31',

'validation_start': '2022-01-01',

'validation_end': '2024-12-31',

'train_ratio': 0.70,

'val_ratio': 0.15,

'test_ratio': 0.15,

'random_seed': 42

},

\# Knowledge graph configuration

'knowledge_graph': {

'embedding_dim': 128,

'margin': 1.0,

'num_negative_samples': 10,

'kg_learning_rate': 0.001,

'kg_epochs': 100

},

\# Model architecture

'model': {

'input_dim': 304,

'hidden_dim': 256,

'num_classes': 3,

'num_layers': 3,

'num_heads': 8,

'num_meta_paths': 6,

'kg_dim': 128,

'dropout': 0.3

},

\# Training configuration

'training': {

'learning_rate': 0.001,

'weight_decay': 1e-4,

'batch_size': 128,

'max_epochs': 200,

'early_stopping_patience': 20,

'label_smoothing': 0.1,

'lambda1': 0.1, # Reconstruction loss weight

'lambda2': 0.05, # Link prediction loss weight

'kg_unfreeze_epoch': 50,

'kg_finetune_lr_ratio': 0.1

},

\# Meta-paths

'meta_paths': [

'R-P-R', # Researcher-Publication-Researcher

'R-I-R', # Researcher-Institution-Researcher

'R-P-C-P-R', # Researcher-Publication-Citation-Publication-Researcher

'R-T-R', # Researcher-Topic-Researcher

'R-F-R', # Researcher-Funding-Researcher

'R-P-T-P-R'	# Researcher-Publication-Topic-Publication-Researcher

],

\# Innovation potential labeling

'labeling': {

'high_potential_percentile': 85, # Top 15%

'low_potential_percentile': 25, # Bottom 25%

'composite_weights': {

'h_index_increment': 0.30,

'citation_growth': 0.25,

'collaboration_expansion': 0.20,

'breakthrough_frequency': 0.15,

'topic_diversity_change': 0.10

}

},

\# Evaluation

'evaluation': {

'metrics': ['accuracy', 'precision', 'recall', 'f1', 'auc_roc', 'ndcg@10'],

'n_bootstrap': 1000,

'confidence_level': 0.95,

'calibration_bins': 10

}

}

\# Relation type mapping

RELATION_TYPES = {

0: 'authorship',

1: 'collaboration',

2: 'citation',

3: 'affiliation',

4: 'mentorship',

5: 'project_participation',

6: 'topic_engagement'

}

\# Entity type mapping

ENTITY_TYPES = {

0: 'researcher',

1: 'publication',

2: 'institution',

3: 'research_topic',

4: 'funding_project'

}

\# Career stage definitions

CAREER_STAGES = {

'early': (0, 5), # 0-5 years since first publication

'mid': (6, 12), # 6-12 years

'senior': (13, 50) # >12 years

}

\# Discipline codes

DISCIPLINES = {

'CS': 'Computer Science',

'ENG': 'Engineering',

'BIO': 'Life Sciences',

'PHY': 'Physics'

}
